# Acute and long-term effects of hip thrust training on athletic performance: a systematic review and meta-analysis

**DOI:** 10.7717/peerj.20785

**Published:** 2026-02-27

**Authors:** Shengfa Lin, Mengna Cheng, Xiaolan Yi, Yuhao Li, Ruidong Liu

**Affiliations:** 1 Sports Coaching College, Beijing Sport University, Beijing, China; 2 Key Laboratory of Sport Training of General Administration of Sport of China, Beijing Sport University, Beijing, China

**Keywords:** Hip thrust, Acute effect, Chronic effect, Performance, Force-vector training, Exercise specificity

## Abstract

**Background:**

The hip thrust (HT) is a popular exercise, but its transfer to athletic performance is debated. We aimed to quantify the acute and long-term effects of HT training on performance.

**Methods:**

Following PRISMA guidelines, a comprehensive search of six electronic databases was conducted to identify randomized controlled trials. Data from 20 studies assessing the effects of HT training on strength, acceleration, and jump performance were synthesized using random-effects models to calculate pooled Hedges’ g effect sizes (ES).

**Results:**

Acutely, the HT induced a moderate post-activation performance enhancement on sprinting (ES = 0.55), dependent on protocol (*e.g.*, multiple sets, ≥4 min recovery). Long-term, HT training significantly improved its own specific strength (ES = 0.53) but failed to transfer to squat strength or jumping performance. While small transfers to change of direction (ES = 0.25) were found, isolated HT training produced borderline - significant sprint transfer (ES = 0.22, *p* = 0.05) when excluding combined-training protocols, suggesting minimal independent benefit for sprint acceleration. The overall certainty of evidence was ‘Low’ to ‘Very Low’ due to high risk of bias.

**Conclusions:**

The HT effectively builds exercise-specific strength, but these gains fail to transfer to squat strength or jumping. Acutely, it offers moderate, protocol-dependent PAPE on sprinting. Long-term, its independent sprint contribution is minimal and borderline-significant (per sensitivity analysis). Based on ‘Low’ to ‘Very Low’ evidence certainty, the HT is a valuable complement, not a replacement, for a complete athletic program.

**Registration:**

This review was registered on the OSF platform, registration number https://doi.org/10.17605/OSF.IO/AYFK3.

## Introduction

The development of lower-body strength and power is a fundamental objective in athletic conditioning, as these qualities underpin critical performance characteristics such as sprinting, jumping, and changing direction (Freitas et al., 2019; Loturco et al., 2018). Central to these actions is the hip extensor muscle group, which collectively serves as the primary driver of hip extension. This group includes several key muscles, most notably the gluteus maximus, the hamstring complex (biceps femoris long head, semitendinosus, semimembranosus), and the adductor magnus (ischial portion) (Beardsley & Contreras, 2014). The relative contribution of these muscles can vary based on joint angle, external load, and the nature of the task (Schoenfeld, 2010). Consequently, identifying optimal training methods to enhance the function of these prime movers is of paramount interest to strength and conditioning professionals, clinicians, and researchers alike.

The hip thrust (HT) has gained significant popularity as an exercise to train the hip extensors, with a specific emphasis on the gluteal musculature (Contreras, Cronin & Schoenfeld, 2011). Its widespread adoption is based on two distinct biomechanical characteristics that theoretically enhance its functional transfer to athletic movements: the horizontal force vector and the demand for maximal hip extension (Brazil et al., 2021). Firstly, the HT is a horizontally-oriented exercise, meaning the external load is primarily resisted in the anterior-posterior direction. This mechanical signature aligns with the “force-vector hypothesis”, positing that HT-induced strength gains should preferentially transfer to tasks requiring high maximal horizontal force production, such as linear acceleration. Secondly, the HT is unique in maintaining a dominant hip extensor moment even as the hip joint approaches full extension, a joint angle critical for propulsive ground reaction forces during maximal sprinting. Additionally, the bent-knee position often used in the HT reduces the mechanical advantage of the hamstrings (active insufficiency), thus potentially increasing the relative contribution of the gluteus maximus to the hip extension torque (Contreras, Cronin & Schoenfeld, 2011; Williams et al., 2021).

Initial research validating the HT focused heavily on its capacity to elicit high levels of neuromuscular activation. A substantial body of evidence, including multiple systematic reviews, has consistently demonstrated that the HT generates greater gluteus maximus electromyographic (EMG) activity compared to the SQ and other common lower-body exercises (Araújo et al., 2023; Krause Neto et al., 2020; Krause Neto, Vieira & Gama, 2019). This high level of acute muscle activation was widely interpreted as evidence of the HT’s superior potential for inducing gluteal hypertrophy and strength gains. However, this paradigm is currently being challenged, as the correlation between acute EMG amplitude and long-term muscle growth is debated (Vigotsky et al., 2022). For instance, some longitudinal studies show that volume-equated HT and SQ training programs can yield similar hypertrophic results in the hip extensors (Plotkin et al., 2023), creating a critical distinction: while the HT may be superior for acutely activating the gluteus maximus, this does not automatically translate into superior long-term morphological adaptations. Therefore, the degree to which HT training ultimately translates into superior functional performance outcomes remains the key unresolved question.

The functional transfer of gains from HT training to athletic performance is highly debated, with the existing literature presenting a fragmented and contradictory picture that necessitates a dual-focus evaluation across acute potentiation and chronic adaptation. The need to examine both is crucial, as they reflect different mechanisms: acute effects indicate the exercise’s potential for short-term neuromuscular potentiation (post-activation performance enhancement, PAPE), utilizing existing strength for an immediate performance boost, while chronic effects reflect its value as a long-term stimulus for lasting morphological and functional adaptations. Regarding acute effects, the HT is often used as a conditioning activity (CA) to induce PAPE, with some studies reporting significant, though inconsistent, improvements in subsequent short-distance sprint performance (Dello Iacono, Padulo & Seitz, 2017; Dello Iacono & Seitz, 2018). However, the evidence for chronic adaptations is highly equivocal; while some long-term interventions have shown that HT training can enhance acceleration sprint speed and exercise-specific strength (Contreras et al., 2017; Zweifel et al., 2017), other well-controlled studies have failed to demonstrate a significant advantage over traditional exercises for improving acceleration, Change of Direction (COD), or jump performance (Jarvis et al., 2019; Lin et al., 2017). These divergent findings are likely compounded by methodological differences in participant training experience, specific loading parameters (*e.g.*, higher loads may reduce power transference), and performance test selection, underscoring the complexity of the HT’s contribution to athletic training.

Given the widespread implementation of the HT in training programs, alongside the persistent uncertainty and contradictory findings regarding its functional transfer, a comprehensive synthesis of the evidence has become critically necessary. While previous systematic reviews have qualitatively discussed these inconsistencies, the field lacks a quantitative synthesis capable of resolving them. Specifically, a differentiation is needed between the HT’s acute effects, which are defined by their utility for PAPE protocols, and its long-term effects, which relate to its value as a chronic stimulus for athletic adaptation. Therefore, this study provides the first systematic review and meta-analysis to quantify both the acute and long-term effects of the HT on foundational athletic parameters, specifically strength, linear acceleration, COD speed, and jump performance. Through this dual-focus approach, this study aims to resolve existing contradictions in the literature and provide clear, evidence-based recommendations for athletes and coaches on the practical application and expected outcomes of incorporating the HT into a training regimen.

## Materials & Methods

This systematic review and meta-analysis was conducted in accordance with the Preferred Reporting Items for Systematic Reviews and Meta-Analyses (PRISMA) guidelines (Page et al., 2021). The study was registered on the Open Science Framework (OSF; registration: https://doi.org/10.17605/OSF.IO/AYFK3). We acknowledge that PROSPERO registration, the preferred platform for systematic reviews of healthcare interventions, was not completed prior to study commencement.

### Search strategy

A comprehensive search was conducted across multiple electronic databases, including PubMed, Web of Science, Scopus, CINAHL, MEDLINE, and SPORTDiscus, from their inception up to August 10, 2025. The database-specific search timeframes were: PubMed (1966), Web of Science (1900), Scopus (1960), CINAHL (1981), MEDLINE (1966), and SPORTDiscus (1949) up to August 2025. All searches were limited to English-language publications. The search strategy employed Boolean operators to combine two primary keyword categories. The first category consisted of intervention keywords, such as “hip thrust”, “barbell hip thrust”. The second category included a broad range of outcome keywords. These keywords covered aspects of strength (“strength”, “1RM”), speed and agility (“sprint”, “change of direction”, “COD”, “agility”), jumping ability (“vertical jump”, “VJ”, “Countermovement Jump”, “CMJ”), acute responses (“acute”, “conditioning activity”, “post activation potentiation”, “PAPE”, “PAP”), muscle activation (“EMG”, ”muscle activation”), and hypertrophy (“muscle thickness”, “cross - sectional area”). This comprehensive search strategy was designed to ensure no relevant performance-focused studies were missed. In addition to the electronic database search, a comprehensive manual search was conducted to identify additional studies. This process involved both a backward and a forward citation search. The backward search consisted of manually screening the reference lists of all included articles and relevant systematic reviews. For the forward search, the Web of Science databases were used to identify all articles that had cited the included studies. The complete search strategy syntax for each database, is provided in Table S1. The number of records retrieved per database was: (PubMed: 106; Web of Science: 165; Scopus: 152; CINAHL: 59; MEDLINE: 108; SPORTDiscus: 106).

### Inclusion and exclusion criteria

Studies were considered eligible for this systematic review and meta-analysis if they met a predefined set of criteria, structured according to the Population, Intervention, Comparator, Outcomes, and Study Design (PICOS) framework. Two of the authors (SL and MC) conducted the initial search, removed duplicates and then screened papers against the inclusion criteria. Inter-rater agreement was assessed using Cohen’s kappa statistic, with kappa = 0.92 indicating excellent agreement (interpretation: kappa > 0.75 = excellent, 0.60–0.75 = good, 0.40–0.59 = fair, < 0.40 = poor). The five studies where disagreement occurred were resolved through discussion, with a third author (YL) included in the review process to provide a consensus.

#### Population

Participants were required to be healthy individuals, with no restrictions on age, sex, or training experience. Studies focusing on clinical populations or individuals with recent musculoskeletal injuries were excluded.

#### Intervention

The intervention had to be a training program where the HT served as the primary and foundational lower-body strength exercise. To reflect its application in practice, protocols were included even if they were part of a broader program containing other secondary movements (such as plyometrics, or sprints), as long as the HT remained the central, high-intensity stimulus being systematically compared against a control or alternative intervention. Interventions that combined the HT with confounding variables were excluded, such as nutritional supplements or the concurrent use of other primary lower-body strength exercises (*e.g.*, the SQ) within the same experimental group.

#### Comparator

For studies investigating acute effects, a within-subject comparator was required, such as a pre-test *vs.* post-test design or a randomized crossover design. For studies investigating chronic (long-term) effects, a control group was required. This could be a passive control group or an active control group performing alternative exercises (*e.g.*, SQ or deadlift).

#### Outcomes

Studies must have reported at least one direct measure of athletic performance, including strength (*e.g.*, SQ strength), speed (*e.g.*, linear acceleration sprint time over a defined distance), COD speed (*e.g.*, performance time in a standardized test like the *T*-test or 505 test), or jump performance. Studies reporting only physiological outcomes (*e.g.*, EMG, muscle thickness) were excluded.

#### Study design

Eligible studies included within-subject repeated measures or crossover trials for acute interventions, and randomized controlled trials (RCTs) for chronic interventions (≥4 weeks duration) (Lin et al., 2025). Studies had to be published in English. Studies were excluded if they were review articles, meta-analyses, case reports, editorials, conference abstracts, dissertations, or books.

### Methodological assessment

The methodological quality and risk of bias of each included study were independently assessed by two reviewers ((SL) and (MC)) using two complementary tools. First, to assess overall external validity and reporting quality, all studies were evaluated using the Physiotherapy Evidence Database (PEDro) scale. The PEDro scale is a valid 10-point tool (De Morton, 2009; Maher et al., 2003), with quality categorized as: ‘excellent’ (9–10), ‘good’ (6–8), ‘fair’ (4–5), or ‘poor’ (<4) (Cashin & McAuley, 2020). Second, to provide a more rigorous and detailed assessment of internal validity, all RCTs included in the quantitative synthesis were assessed using the Risk of Bias 2 (RoB 2) tool (Sterne et al., 2019). Any discrepancies in the PEDro scores or RoB 2 judgments between the two primary reviewers were resolved through discussion and, if necessary, consultation with a third reviewer ((YL)) to reach a final consensus.

### Data extraction

Two independent reviewers (SL and MC) systematically extracted relevant data from the included studies using a standardized data extraction form. Any discrepancies were resolved through discussion or consultation with a third reviewer (YL) to achieve consensus. The extracted information included: (1) study author and publication year; (2) participant characteristics (sample size, age, sex, sport background, and resistance training experience); (3) details of the acute intervention (volume, intensity, and rest interval duration); (4) details of the long-term intervention (training frequency, duration, volume, and intensity); and (5) outcome data, specifically the mean and standard deviation (SD) of performance measures for all groups at pre- and post-intervention time points. In cases of missing or incompletely reported data, the corresponding authors of the original studies were contacted for clarification.

### Meta-analysis

All statistical analyses were conducted using Stata/MP (version 17.0; StataCorp LLC, College Station, TX, USA), with the level of statistical significance set at *p* < 0.05. The effect size (ES) was calculated as Hedges’ g, which provides a standardized mean difference adjusted for small sample bias. For acute effect studies, the ES was based on the performance change from pre- to post-intervention (Tillin & Bishop, 2009). For long-term interventions, Hedges’ g was calculated from the pre- and post-intervention means and SDs of the HT and control groups (Chandler et al., 2019). The magnitude of the ES was interpreted according to Cohen’s criteria as trivial (ES < 0.20), small (0.20 ≤ ES < 0.50), moderate (0.50 ≤ ES < 0.80), or large (ES ≥ 0.80) (Cohen, 1992). Due to the anticipated variability in study populations and intervention protocols, a random-effects model (DerSimonian and Laird) was employed for all meta-analyses to pool the ES estimates (Chandler et al., 2019). Statistical heterogeneity among studies was assessed using the *I*^2^ statistic, with values of <25%, 25–75%, and >75% considered to represent low, moderate, and high levels of heterogeneity, respectively (Higgins & Thompson, 2002). The risk of publication bias was evaluated for each outcome by visually inspecting the symmetry of its corresponding contour-enhanced funnel plot. All funnel plots are available in File S1. For meta-analyses including 10 or more studies, this visual assessment was supplemented with a formal statistical analysis using Egger’s regression test (Egger et al., 1997; Sterne et al., 2011). A *p*-value less than 0.05 was considered indicative of statistically significant publication bias. In cases where significant bias was detected, a sensitivity analysis was conducted using the trim and fill method to estimate an adjusted effect size that accounted for the potential impact of missing studies (Duval & Tweedie, 2000). While primary studies require prospective power calculations, meta-analyses also benefit from adequacy assessment (Mainer Pardos et al., 2024). With *k* = 7–13 studies per outcome, our analyses met the minimum threshold (k ≥ 4) for random-effects meta-analysis, though moderator analyses with *k* = 2–3 per subgroup should be interpreted cautiously due to limited statistical power.

### Sensitivity analysis

We conducted several sensitivity analyses to evaluate the robustness of our pooled findings. First, to isolate the standalone effect of the intervention, an analysis was performed by excluding studies that utilized combined training protocols (*i.e.,* those that mixed the primary hip thrust intervention with other auxiliary exercises). Second, to investigate the influence of potential outliers on outcomes with high heterogeneity, we conducted analyses by systematically excluding studies identified as having large deviations from the overall pooled effect. The results of these analyses were then compared to the primary analysis to determine the impact of these studies on the overall effect size and heterogeneity (*I*^2^).

### Certainty of evidence

To evaluate the overall certainty of the evidence for each main outcome (*e.g.*, sprint, jump, and strength performance), we used the Grading of Recommendations, Assessment, Development and Evaluations (GRADE) framework (Guyatt et al., 2011). Following established guidelines, the certainty of evidence was assessed across five domains: (1) risk of bias (from our RoB 2 analysis), (2) inconsistency (heterogeneity), (3) indirectness, (4) imprecision, and (5) publication bias. The final certainty for each outcome was rated as ‘High’, ‘Moderate’, ‘Low’, or ‘Very Low’ (Zhang et al., 2019). Any discrepancies in the GRADE ratings between the two primary reviewers ((SL) and (MC)) were resolved through discussion and, if necessary, consultation with a third reviewer (XY).

### Moderator analysis

To investigate potential sources of heterogeneity, a series of pre-planned moderator analyses were conducted. A subgroup analysis was performed for any given moderator only if each category contained at least two studies, ensuring a valid comparison. The selection of categories and cut-off points for these variables was based on several principles. For variables with established classifications, such as age (<18 *vs.* ≥18 years), sex, and training background (trained *vs.* untrained), logically defensible and commonly accepted groupings were used. In cases lacking a clear theoretical basis, a data-driven median split of the included studies determined the thresholds for variables like intervention duration (8 weeks) and frequency (three times per week). Furthermore, to ensure consistency and comparability with existing evidence, other moderators such as training volume (single set *vs.* multiple sets), and recovery duration (*e.g.*, Immediate “<4 min”, short “4–7 min”, moderate “8–10 min”, long “>10 min”) were categorized using thresholds established in prior systematic reviews and meta-analyses (Seitz & Haff, 2016; Wilson et al., 2013). Additionally, to assess the impact of methodological quality, a subgroup analysis was performed based on the RoB 2 risk of bias ratings, comparing studies judged to have ‘some concerns’ against those at ‘high risk’ of bias. Intervention intensity was dichotomized at 85% of one-repetition maximum (1RM). This threshold was chosen based on established resistance training guidelines, as it represents the approximate lower limit for training primarily aimed at developing maximal strength, distinguishing it from training focused on muscular hypertrophy (National Strength Conditioning Association, 2021).

## Results

### Study selection

**Figure 1:**
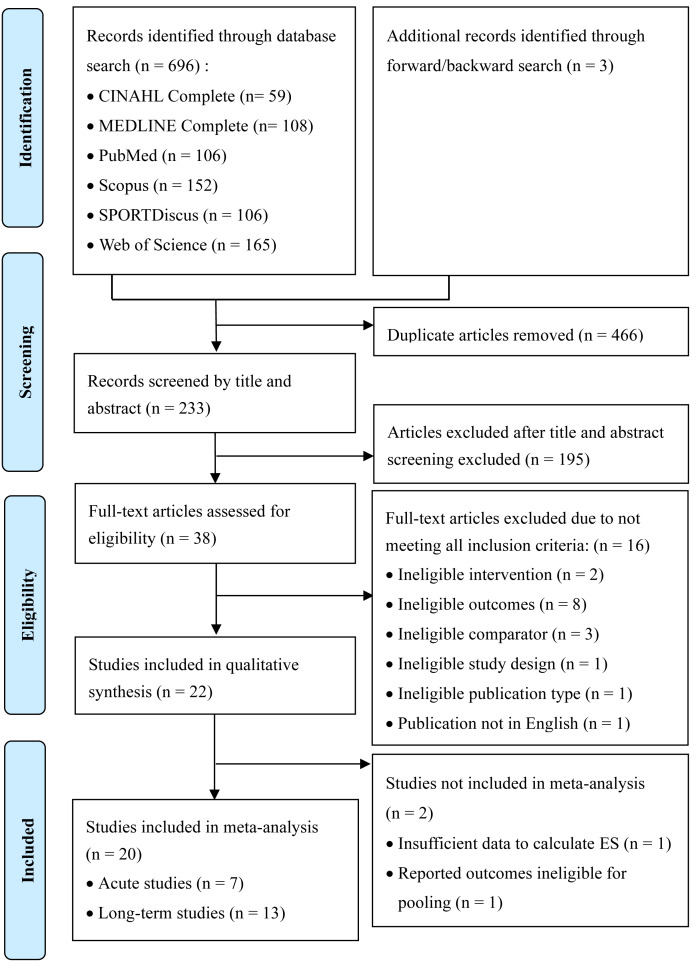
Study selection flowchart.

The initial search yielded a total of 699 records, with 696 identified through electronic database searches and an additional three records identified through manual forward and backward citation searching. After the removal of 466 duplicate records, 233 unique articles proceeded to the title and abstract screening phase. During this screening, 195 records were excluded as they did not meet the review’s scope, leaving 38 articles for full-text eligibility assessment. Following a thorough full-text review, 16 of these articles were further excluded. The primary reasons for exclusion at this stage were: reporting ineligible outcomes (*n* = 8), not utilizing a HT intervention (*n* = 2), absence of a control group (*n* = 3), ineligible study design (*i.e.,* non-randomized trial) (*n* = 1), ineligible publication type (*n* = 1), and publication not in English (*n* = 1). Ultimately, a total of 22 studies met the inclusion criteria. All 22 studies were included in the qualitative synthesis, while 20 of these studies provided sufficient data for inclusion in the quantitative synthesis (meta-analysis). The detailed flowchart of the entire study selection process is presented in Fig. 1.

### Methodological assessment of included studies

**Figure 2:**
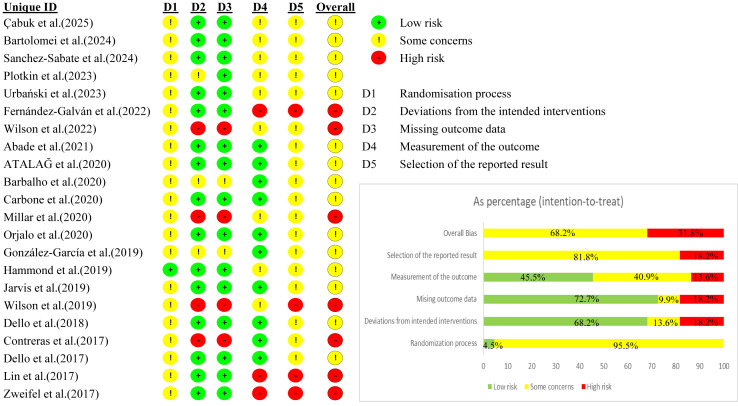
Risk of bias in the included studies.

A total of 22 studies met the inclusion criteria and were subjected to methodological quality and risk of bias assessment using both the PEDro scale and the RoB 2 tool. The PEDro assessment revealed that the overall quality of the included RCTs ranged from ‘fair’ to ‘good’ (total scores from 4 to 7 out of 10). The majority of the studies, fourteen in total (63.6%), were classified as ‘good’ quality (score 6–7). The remaining eight studies (36.4%) were rated as ‘fair’ quality (score 4–5). A detailed analysis of individual items (Table 1) revealed consistent methodological strengths, including appropriate random allocation (100%) and baseline comparability (100%). However, significant limitations were consistently noted in other areas, particularly blinding of subjects (0/22 studies) and therapists (0/22 studies), as well as allocation concealment (1/22 studies, 4.5%). Furthermore, the RoB 2 assessment (Fig. 2) revealed significant concerns regarding the internal validity of the included trials. Overall, no studies (0%) were judged to be at a ‘low risk’ of bias. The vast majority of studies were judged to have ‘some concerns’ (15 studies, 68.2%) or were at a ‘high risk’ of bias (seven studies, 31.8%). The detailed ‘traffic light’ plot and summary of the RoB 2 assessment are presented in Fig. 2.

**Table 1:** PEDro scores of the included studies.

Study	1^*^	2	3	4	5	6	7	8	9	10	11	Total
Çabuk & İnce (2025)	1	1	0	1	0	0	0	1	0	1	1	5
Bartolomei et al. (2024)	1	1	0	1	0	0	0	1	1	1	1	6
Sánchez-Sabaté et al. (2024)	1	1	0	1	0	0	0	1	1	1	1	6
Plotkin et al. (2023)	1	1	0	1	0	0	0	1	0	1	1	5
Urbanski et al. (2023)	1	1	0	1	0	0	0	1	1	1	1	6
Fernandez-Galvan et al. (2022)	1	1	0	1	0	0	0	1	1	1	1	6
Wilson et al. (2022)	1	1	0	1	0	0	0	0	0	1	1	4
Abade et al. (2021)	1	1	0	1	0	0	0	1	1	1	1	6
Atalag et al. (2020)	1	1	0	1	0	0	0	1	1	1	1	6
Barbalho et al. (2020)	1	1	0	1	0	0	0	1	1	1	1	6
Carbone et al. (2020)	1	1	0	1	0	0	0	1	1	1	1	6
Millar et al. (2020)	1	1	0	1	0	0	0	0	0	1	1	4
Orjalo, Callaghan & Lockie (2020)	1	1	0	1	0	0	0	1	1	1	1	6
González-García et al. (2019)	1	1	0	1	0	0	0	1	0	1	1	5
Hammond et al. (2019)	1	1	1	1	0	0	0	1	1	1	1	7
Jarvis et al. (2019)	1	1	0	1	0	0	0	1	1	1	1	6
Wilson et al. (2019)	1	1	0	1	0	0	0	0	0	1	1	4
Dello Iacono & Seitz (2018)	1	1	0	1	0	0	0	1	1	1	1	6
Contreras et al. (2017)	1	1	0	1	0	0	1	1	0	1	1	6
Dello Iacono, Padulo & Seitz (2017)	1	1	0	1	0	0	0	1	1	1	1	6
Lin et al. (2017)	1	1	0	1	0	0	0	1	1	0	1	5
Zweifel (2017)	1	1	0	1	0	0	0	1	1	0	0	4
Summary	100%	100%	4.5%	100%	0%	0%	4.5%	86.4%	68.2	90.9%	95.5%	–

**Notes.**

* The first criterion was excluded for the calculation of the PEDro score.

### Study characteristics

A total of eight studies that investigated acute effects were identified (Table 2), of which seven were included in the quantitative meta-analysis. The remaining one acute study (Orjalo, Callaghan & Lockie, 2020) was included only in the qualitative synthesis as it reported a COD outcome measure that could not be pooled. The eight acute studies included a total of 154 participants. These participants were predominantly male (six studies included males, two were mixed-sex) with mean ages ranging from 15.6 to 23.3 years. Most were experienced athletes, with participants from soccer, tennis, rugby, handball, and other field or court sports. The HT protocols used for PAPE typically involved low-volume (1–3 sets of 3–10 repetitions), high-intensity loads, frequently at or above 84% 1RM. A wide range of recovery intervals were investigated, from 15 s to 16 min post-intervention.

**Table 2:** Characteristics of included long term intervention studies.

Study	Sample characteristics					HT protocol			Outcomes	Effect size range (Hedge’s g)
	*n*	Age (yrs)^*^	Sex	Sport	Resistance training experience	Volume (sets × reps)	Intensity (%1RM)	Rest interval		
Çabuk & İnce (2025)	13	15.6	M	Soccer	2.55 ± 0.75 yrs	1 × 3 & 1 × 6	84%1RM & 60%1RM	7 min	10 m, 20 m and 30 m sprint	0.18∼0.65
Urbanski et al. (2023)	12	18∼19	M	Soccer	>2 yrs	2 × 3	90%1RM	5 min	CMJ and SLJ	0.07∼0.38
Fernandez-Galvan et al. (2022)	19	15.6	M	Tennis	None	1 × 3 & 1 × 7	85%1RM & 60%1RM	4min	5 m, 10 m and 30 m sprint	0.05∼0.43
Atalag et al. (2020)	17	21.7	Mix	Healthy	5.47 ± 2.00 yrs	1 × 3	90%1RM	8 min	CMJ, 18.3 m, and 36.6 m sprint	−0.16∼0.31
Carbone et al. (2020)	17	22.1	M	Rugby	>2 yrs	3 × 3, 3 min rest between sets	85%1RM	8 min	5 m and 10 m sprint	−0.08∼0.06
Orjalo, Callaghan & Lockie (2020)	40	23.3	Mix	Field or court sports	>1 yrs	3 × 5, 2 min rest between sets	85% 1RM	4, 8, 12 and 16 min	505 test	0.19∼0.32
Dello Iacono & Seitz (2018)	18	19.3	M	Soccer	>3 yrs	3 × 6 & 3 × 8	85%1RM & OPL%1RM	15 s, 4 and 8 min	5 m, 10 m and 20 m sprint	−0.29∼2.14
Dello Iacono, Padulo & Seitz (2017)	18	19.8	M	Handball	>3 yrs	3 × 6 & 3 × 10	85%1RM & 50%1RM	15 s, 4 and 8min	10 m and 15 m sprint	−1.55∼2.60

**Notes.**

%1RM percentages of one-repetition maximum 1RM one-repetition maximum CMJ countermovement jump COD change of direction F female HT hip thrust M male Mix both male and female *n* sample sizes OPL Optimal Power load reps repetitions SLJ standing long jump wk week yrs years

Age is reported as mean where provided by the source study, otherwise, the reported range is used.

A total of 14 long-term studies were identified (Table 3). Of these, 13 provided sufficient data for the quantitative meta-analysis, while one study was included only in the qualitative synthesis due to insufficient data for ES calculation (Plotkin et al., 2023). The 14 long-term intervention studies involved a total of 328 participants. The participant age varied, with mean ages reported from 15.3 to 27.5 years and some studies including participants up to 45 years old. These studies included male-only (*n* = 7), female-only (*n* = 2), and mixed (*n* = 5) cohorts. Participants came from a range of athletic backgrounds, including soccer, basketball, rugby, and baseball, as well as generally healthy or athletic individuals. Training experience varied, with seven studies involving participants with at least one year of resistance training experience and seven studies using untrained or novice individuals. The training interventions ranged from 4 to 20 weeks in duration, with training frequencies between one and four sessions per week. Most long-term studies utilized periodized training models, with intensities prescribed using percentages of 1RM (%1RM), RM targets, or repetitions in reserve. Additionally, in some of these interventions, the HT was not used in isolation but was employed as the core resistance exercise, combined with other auxiliary exercises to form a comprehensive training protocol (Bartolomei et al., 2024; Sánchez-Sabaté et al., 2024).

**Table 3:** Characteristics of included long term intervention studies.

Study	Sample characteristics					HT interventions				Outcomes
	*n*	Age^*^	Sex	Sport	Resistance training experience	Freq (wk^−1^)	Dura (wk)	Volume (sets × reps)	Intensity (%1RM, RM, RIR)^#^	
Bartolomei et al. (2024)	HTG = 10 SQG = 9	25.9∼26.9	Mix	Healthy	7.6 ± 6.0 years	4	6	Periodized: 5 × 8 → 3 × 3	1 RIR	1RM-HT, 1RM-SQ, SLJ, CMJ, and 20 m sprint
Sánchez-Sabaté et al. (2024)	HTG = 9 SQG = 9	15.9	M	Basketball	2 years	2	8	3 × 6–3 × 8	30–45% 1RM	CMJ, V-cut test, 5 m, 10 m, and 20 m sprint
Plotkin et al. (2023)	HTG = 18 SQG = 16	22∼24	Mix	Healthy	<1 day /wk	2	9	3 sets → 6 sets	8–12 RM to failure	3RM-SQ and 3RM-HT
Wilson et al. (2022)	HTG = 11 SQG =11 DLG = 11	18∼45	M	Healthy	None	2	6	3 × 8 → 5 × 4	75% → 85% 1RM	CMJ and SLJ
Abade et al. (2021)	HTG = 8 SQG = 8 CG = 8	16.5	M	Soccer	None	1	20	3 × 10-8 → 3 × 4–6	10-8 → 4–6 RM	SJ, CMJ, SLJ, 10 m, and 20 m sprint
Barbalho et al. (2020)	HTG = 10 SQG = 12	27.5	F	Healthy	5.1 ± 0.7 years	1	12	6 sets per wk	Non-linear periodization	1M-HT and 1RM-SQ
Millar et al. (2020)	HTG = 6 SQG = 8	15.3	F	Soccer	None	2	6	3 × 8 → 3 × 4	3RM-based, ∼10% weekly load increase	3RM-HT, 3RM-SQ, CMJ, SLJ, 36.6 m sprint, and Pro-agility
González-García et al. (2019)	HTG = 8 SQG = 8 CG = 8	16.8	F	Soccer	None	2	7	4 × 12 → 4 × 4	60% → 90% 1RM	CMJ, *T*-test, 10 m, and 2 0m sprint
Hammond et al. (2019)	HTG = 7 SQG = 7	22.4	M	Healthy	>6 months	2	4	3 sets	80%1RM to failure	1RM-HT and 1RM-SQ
Jarvis et al. (2019)	HTG = 11 CG = 10	27	Mix	Healthy	>1 year	2	8	5 × 5	85% 1RM	1RM-HT, 10 m, and 40 m sprint
Wilson et al. (2019)	HTG = 11 SQG = 11 DLG = 11	18∼45	M	Healthy	None	2	6	3 × 8 → 5 × 4	75% → 85% 1RM	1RM-HT and 1RM-SQ
Contreras et al. (2017)	HTG = 13 SQG = 11	14∼17	M	Rugby and rowing	1 year	2	6	4 × 12 → 4 × 6	12 → 6 RM	3RM-HT, 3RM-SQ, CMJ, SLJ, 10 m, and 20 m
Lin et al. (2017)	HTG = 10 CG = 10	19.9	M	Baseball	>1 year	3	8	4 × 20 → 4 × 6	50% → 90% 1RM	1RM-SQ, 3RM-HT, CMJ, SLJ, and 30 m sprint
Zweifel et al. (2017)	HTG = 8 SQG = 8 DLG = 8 CG = 4	22.1	Mix	Athletic background	>1 year	3	6	4 × 6–8 → 5–6 × 2–4	72–80% → 25–55%1RM	1RM-HT, 1RM-SQ, CMJ, SLJ, 9.14 m and 36.6 m sprint

**Notes.**

%1RM percentages of one-repetition maximum 1RM one-repetition maximum CG control group CMJ countermovement jump COD change of direction DLG deadlift group Dura duration F female Freq frequency HT hip thrust HTG hip thrust group M male Mix both male and female N/A not applicable *n* sample sizes reps repetitions RIR repetitions in reserve RM repetition maximum SJ squat jump SLJ standing long jump SQ squat SQG squat group wk week

* Age is reported as mean where provided by the source study, otherwise, the reported range is used.

# %1RM, RM, and RIR are presented as reported in the primary studies and are considered equivalent methods for prescribing and monitoring training intensity. While methods like ‘Non-linear periodization’ and ‘to failure’ approaches differ from percentage-based prescriptions in their execution, they are included as equivalent methods for prescribing and monitoring resistance training intensity.

### Meta-analysis of acute performance effects

#### Acute effect on linear acceleration sprint performance

**Figure 3:**
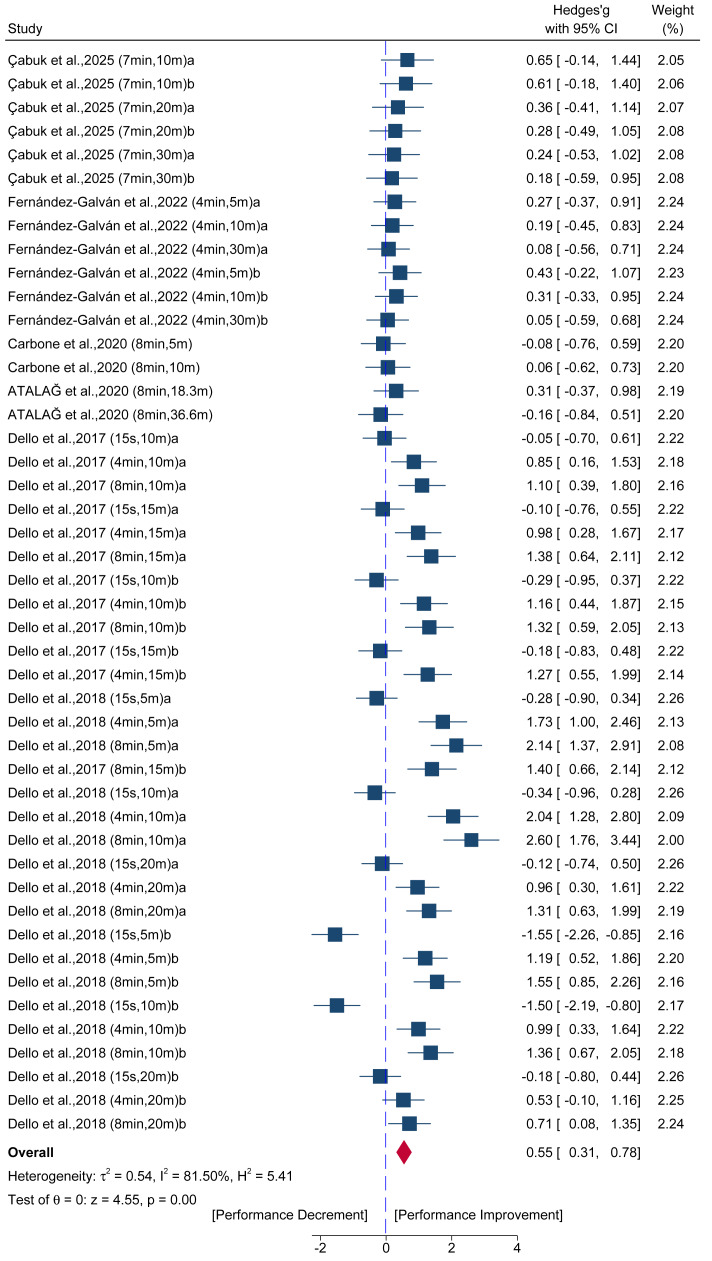
Forest plot of the acute pre- to post-intervention effects of a hip thrust (HT) conditioning activity on linear acceleration sprint performance. Squares represent the effect size (Hedges’ *g*) from individual comparisons, with their size proportional to their weight in the meta-analysis. The diamond indicates the pooled effect size with its 95% confidence interval (CI). Effect sizes to the right of the vertical “zero” line indicate a performance improvement post-intervention, while effects to the left indicate a performance decrement. Interpretation of Hedges’ *g* follows Cohen’s conventions: trivial (ES < 0.20), small (0.20 ≤ ES < 0.50), moderate (0.50 ≤ ES < 0.80), and large (ES ≥ 0.80).

Data from 46 comparisons across six studies provided data for acute linear acceleration sprint performance. The results (Fig. 3) showed a significant improvement in sprint performance favoring the HT group over the control group (ES = 0.55; 95% CI [0.31–0.78]; *p* < 0.001; *I*^2^ = 81.50%), with Egger’s test indicating significant publication bias (*p* < 0.001). Visual inspection of the contour-enhanced funnel plot (File S1) confirmed this finding, revealing that the asymmetry was primarily caused by an absence of studies within the non-significant (*p* > 0.10) region of the plot. The trim-and-fill analysis, however, did not impute any missing studies, resulting in an adjusted pooled effect size that was identical to the observed effect (ES = 0.55; 95% CI [0.31–0.78]). This finding suggests the funnel plot asymmetry may be due to factors other than publication bias, such as residual heterogeneity or small-study effects.

#### Acute effect on jump performance

**Figure 4:**
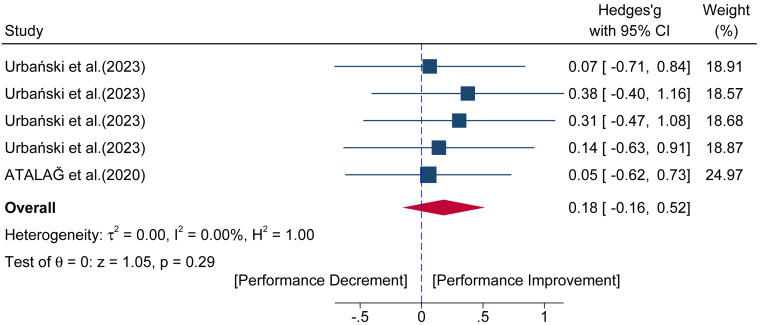
Forest plot of the acute pre- to post-intervention effects of a hip thrust conditioning activity on jump performance. Squares represent the effect size (Hedges’ *g*) from individual comparisons, with their size proportional to their weight in the meta-analysis. The diamond indicates the pooled effect size with its 95% confidence interval (CI). Effect sizes to the right of the vertical “zero” line indicate a performance improvement post-intervention, while effects to the left indicate a performance decrement.

Data from five comparisons across two studies provided data for acute jump performance. The results (Fig. 4) showed no significant difference in jump performance between the HT group and the control group (Hedges’ *g* = 0.18; 95% CI [−0.16–0.52]; *p* = 0.29; *I*^2^ = 0.00%).

### Meta-analysis of long-term training adaptations

#### Long-term effect on strength performance

**Figure 5:**
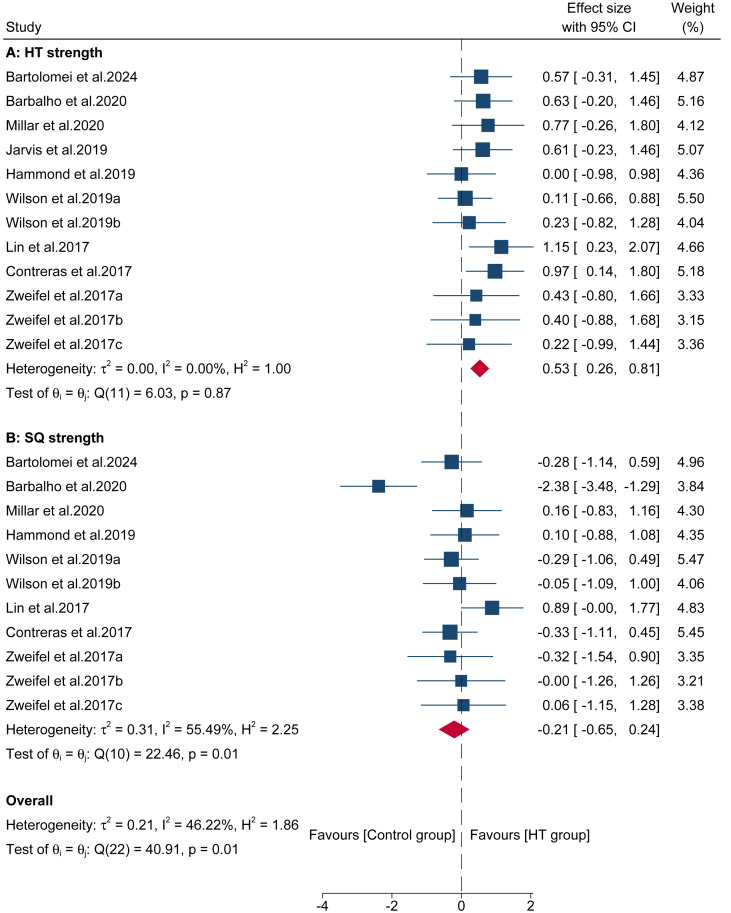
Forest plot comparing the long-term effects of HT *versus* control training on (A) HT strength and (B) squat (SQ) strength. Squares represent the effect size (Hedges’ *g*) from individual comparisons, with their size proportional to their weight in the meta-analysis. The diamond indicates the pooled effect size with its 95% confidence interval (CI). Effect sizes to the right of the vertical “zero” line favor the HT group.

The analysis first examined HT strength, using data from 12 comparisons across 9 studies that involved 195 participants. The results (Fig. 5A) indicated a significant advantage for the HT group compared to the control group (ES = 0.53; 95% CI [0.26–0.81]; *p* < 0.001; *I*^2^ = 0.00%), with Egger’s test indicating no significant publication bias (*p* = 0.54). In contrast, when assessing SQ strength, data from 11 comparisons across eght studies (involving 174 participants) showed no significant difference between the groups (Fig. 5B) (ES = −0.21; 95% CI [−0.65–0.24]; *p* = 0.37; *I*^2^ = 55.49%), with Egger’s test indicating no significant publication bias (*p* = 0.69). After a sensitivity analysis, excluding the study of Barbalho et al. (2020), similar results were found (ES = −0.02; 95% CI [−0.32–0.28]; *p* = 0.90; *I*^2^ = 0.00%).

#### Long-term effect on linear acceleration sprint performance

**Figure 6:**
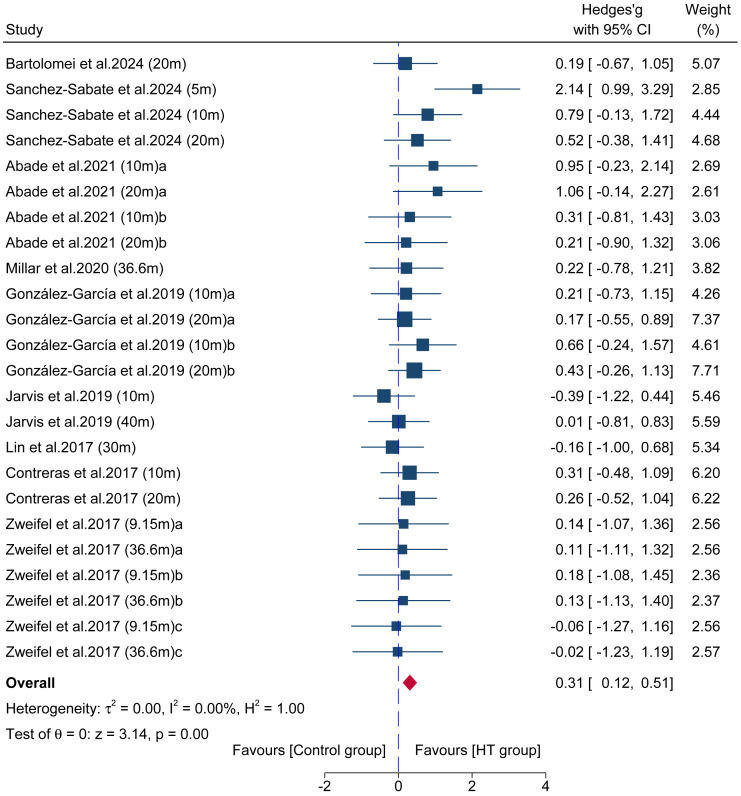
Forest plot comparing the long-term effects of HT *versus* control training on linear acceleration sprint performance. Squares represent the effect size (Hedges’ *g*) from individual comparisons, with their size proportional to their weight in the meta-analysis. The diamond indicates the pooled effect size with its 95% confidence interval (CI). Effect sizes to the right of the vertical “zero” line favor the HT group.

Data from 24 comparisons across nine studies provided data for linear acceleration sprint performance, involving a total of 192 participants. The results (Fig. 6) showed a significant improvement in sprint performance favoring the HT group over the control group (ES = 0.31; 95% CI = 0.12 to 0.51; *p* < 0.001; *I*^2^ = 0.00%), with Egger’s test indicating no significant publication bias (*p* = 0.29).

#### Long-term effect on change of direction speed performance

**Figure 7:**
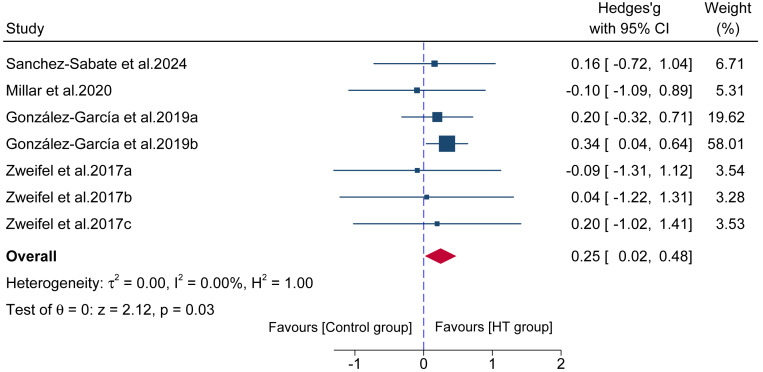
Forest plot comparing the long-term effects of HT *versus* control training on change of direction (COD) speed. Squares represent the effect size (Hedges’ * g*) from individual comparisons, with their size proportional to their weight in the meta-analysis. The diamond indicates the pooled effect size with its 95% confidence interval (CI). Effect sizes to the right of the vertical “zero” line favor the HT group.

Data from seven comparisons derived from four studies provided data for COD speed, involving 85 participants. The results (Fig. 7) showed a significant difference between HT group and control group (ES = 0.25; 95% CI [0.02–0.48]; *p* = 0.03; *I*^2^ = 0.00%).

#### Long-term effect on jump performance

**Figure 8:**
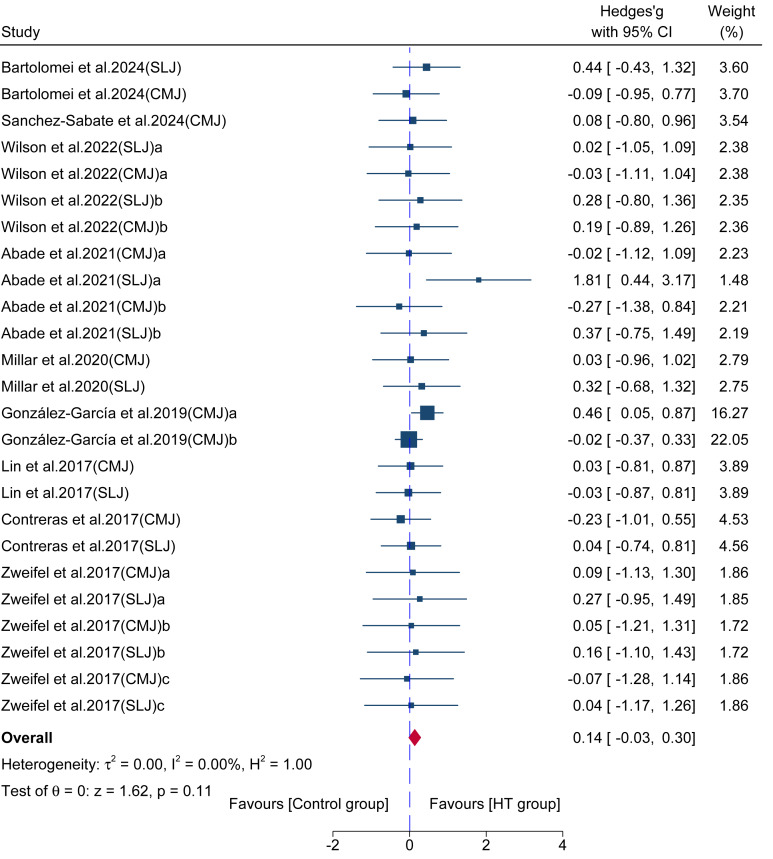
Forest plot comparing the long-term effects of HT *versus* control training on jump performance. Squares represent the effect size (Hedges’ *g*) from individual comparisons, with their size proportional to their weight in the meta-analysis. The diamond indicates the pooled effect size with its 95% confidence interval (CI). Effect sizes to the right of the vertical “zero” line favor the HT group.

Data from 25 comparisons across nine studies provided data for jump performance, involving a total of 204 participants. The results (Fig. 8) showed no significant difference between the HT group over the control group (ES = 0.14; 95% CI [−0.03–0.30]; *p* = 0.11; *I*^2^ = 0.00%), with Egger’s test indicating no significant publication bias (*p* = 0.84).

### Sensitivity analyses

A series of sensitivity analyses were conducted to assess the impact of potential outliers and studies using combined training protocols on the results of the meta-analysis. The results of these analyses are presented in Table S2. For the acute linear acceleration sprint analysis, the study by Dello Iacono & Seitz (2018) was identified as a major outlier. After removing this study, heterogeneity was substantially reduced (from 81.50% to 49.83%), and the pooled ES also decreased (from 0.55 to 0.40). However, the result remained statistically significant (*p* < 0.001), indicating that while this single study contributed to the high heterogeneity and inflated the ES, the overall positive finding remains robust. For the long-term SQ strength analysis, the study by Barbalho et al. (2020) was identified as an outlier. After removing this study, all heterogeneity was eliminated (from 55.49% to 0.00%). This removal also shifted the pooled ES from −0.21 (*p* = 0.37) to a null effect (ES = −0.02, *p* = 0.90), confirming that the original pooled result was strongly influenced by this single study. The analyses excluding studies with combined training protocols revealed that for long-term sprint performance, removing these studies reduced the pooled ES (from 0.31 to 0.22) and rendered the finding borderline significant (*p* = 0.05). For all other chronic outcomes (*e.g.*, HT strength, COD, and jump performance), removing the combined training studies did not meaningfully alter the pooled effect sizes or conclusions.

### Certainty of evidence

The overall certainty of evidence was assessed using the GRADE tool, and the results are presented in Table S3. The GRADE assessment indicated very low certainty for acute linear acceleration sprint performance. Certainty was graded as low for acute jump performance, long-term back squat strength, change of direction speed, long-term jump performance, and long-term linear acceleration sprint performance. This indicates that there is limited confidence in the effect estimates for these outcomes. The certainty of evidence for long-term hip thrust strength was graded as moderate. This suggests a moderate level of confidence in the effect estimate for this outcome, although further research could still have an impact on the estimate.

### Moderator analyses

A moderator analysis was conducted to examine the influence of different variables (Table S4). For the acute effects on linear acceleration sprint, significant differences were found between recovery durations (*p* < 0.001) and between training volumes (*p* = 0.03), where multiple sets showed greater effects than single sets. Additionally, a borderline significant difference was found based on risk of bias (*p* = 0.05), where studies judged to have ‘some concerns’ reported larger effects than those judged at ‘high risk’. For long-term training adaptations, a significant difference was observed in linear acceleration sprint performance between age groups (*p* = 0.01), favoring the <18 years group. No other significant differences were found for any other moderators in the long-term adaptation outcomes. Some moderator analyses were not performed for certain outcomes due to a limited number of available studies.

## Discussion

The results of this meta-analysis serve to reconcile a foundational debate surrounding the HT. For years, a significant body of research has established that the HT elicits superior gluteus maximus EMG activity compared to traditional exercises (Krause Neto et al., 2020; Krause Neto, Vieira & Gama, 2019), creating a plausible hypothesis that it would yield superior performance outcomes. The present meta-analysis, the first to quantitatively synthesize both the acute and long-term effects of the HT, provides a critical, performance-based test of this hypothesis. The main findings reveal a nuanced reality: the HT’s exceptional capacity for acute muscle activation and developing exercise-specific strength does not uniformly translate into superior performance in broader athletic tasks.

Specifically, in terms of acute effects, the HT serves as an effective conditioning activity, inducing a moderate PAPE in subsequent sprint performance (ES = 0.55), though this benefit is highly dependent on recovery duration and training volume, with no significant effect found for jumping. Concurrently, long-term HT training significantly improves its exercise-specific strength (ES = 0.53) and yields small but significant gains in linear acceleration sprint (ES = 0.31) and COD speed (ES = 0.25). However, these benefits do not transfer to SQ strength or jumping performance. Collectively, these findings establish the HT not as a universally superior modality, but as a specialized tool for developing horizontal force production, which must be applied in a context-specific manner.

### Acute effects on performance

A core finding of this study is the moderate and significant effect of the HT as a CA on subsequent linear acceleration sprint performance (ES = 0.55). This aligns with the fundamental principles of PAPE, where a high-intensity conditioning exercise enhances neuromuscular excitability (Chen, Lim & Thapa, 2024; Tillin & Bishop, 2009). This effect likely reflects the HT’s capacity to enhance the rate and magnitude of force development in hip extensors during the ground contact phase of sprinting. Recent kinematic analyses of sprint acceleration demonstrate that powerful hip extension during ground contact is the primary driver of forward propulsion, with peak forces occurring at hip angles similar to those trained during the HT (∼180° hip extension) (Le et al., 2024).

The classic “potentiation-fatigue” trade-off was evident in the recovery duration: insufficient recovery (<4 min) suppressed performance, while adequate recovery (≥4 min) allowed fatigue to dissipate and potentiation to manifest, resulting in significantly improved performance. This temporal optimization was key to managing heterogeneity, as demonstrated by the subgroup analyses: While the overall pooled ES was 0.55 (*I*^2^ = 81.50%), the short recovery window of 4–7 min resulted in an optimal ES of 0.69 (moderate) with a substantially reduced heterogeneity of *I*^2^ = 55.48%. The moderate recovery window of 8-10 min achieved the largest effect size, ES = 1.05 (*I*^2^ = 79.49%), confirming that maximal potentiation is achievable under optimized timing.

Beyond recovery time, training volume was also a significant moderator, with multiple sets (ES = 0.67, *I*^2^ = 86.48%) proving superior to single sets (ES = 0.28, *I*^2^ = 0.00%) for inducing PAPE. This suggests that a greater stimulus is needed to maximize the potentiation response. The effectiveness of the HT in potentiating sprinting once again highlights the force-vector theory, as its horizontal loading pattern precisely primes the neuromuscular pathways required for horizontal acceleration (Williams et al., 2021; Zweifel, 2017). However, despite identifying recovery duration and volume as significant moderators, substantial heterogeneity persisted within subgroups (*e.g.*, *I*^2^ = 79.49% for the 8–10 min recovery subgroup), suggesting additional unmeasured factors contribute to effect variability. Potential sources include individual athlete characteristics such as baseline strength levels, training age, and sport-specific movement patterns (Skorulski et al., 2025), which could not be extracted consistently across studies.

Nonetheless, the sensitivity analysis supports the robustness of the core finding. The study by Dello Iacono & Seitz (2018) was identified as a significant outlier influencing heterogeneity. After systematically removing this single study, the overall effect size remained significant at ES = 0.40 (small to moderate effect), and crucially, heterogeneity was drastically reduced to *I*^2^ = 49.83%. The fact that the ES remained positive and significant while heterogeneity dropped below the 50% threshold strongly indicates that the initial high variability was primarily driven by one influential study, and that the underlying positive effect of HT-induced PAPE is statistically robust against outlier removal.

In stark contrast to its effect on sprinting, the analysis showed no significant acute facilitative effect of the HT on jumping performance (ES = 0.18). This finding is equally explainable by the principle of dynamic correspondence (Verkhoshansky & Verkhoshansky, 2011; Zatsiorsky, Kraemer & Fry, 2020). For the CMJ, the lack of potentiation is due to the mismatch between the horizontal force vector of the HT and the vertical demands of the jump. For the SLJ, despite both being horizontal tasks, the motor patterns are distinct; the SLJ is a ballistic, multi-joint explosive action, whereas the HT is a more controlled, isolated hip extension movement (Zweifel, 2017). This dissimilarity means the HT may not sufficiently prime the specific neuromuscular coordination required for an optimal jump, implying that the magnitude of performance enhancement is highly contingent on the biomechanical specificity between the CA and the subsequent performance task (Abade et al., 2024).

Further nuance is provided by studies included in the qualitative synthesis. For instance, the work of Vurmaz et al. (2025), while excluded from the meta-analysis, offers qualitative support for the temporal dynamics of PAPE, observing progressively faster 20 m sprint times at 4 min and 8 min rest intervals compared to a 15 s interval. Additionally, the study by Orjalo, Callaghan & Lockie (2020) on COD performance found that while the HT did potentiate the 505 test, this improvement was not significantly greater than a passive control condition. The likely reason for this disparity is that an acute PAPE stimulus enhances the foundational physical quality of force production but does not immediately refine the sophisticated neuromuscular coordination and specific movement strategies required for a complex COD task (Neptune, Wright & Van Den Bogert, 1999; Orjalo, Callaghan & Lockie, 2020). This distinction explains why long-term training is effective, as it provides the necessary time for the nervous system to integrate the newly acquired strength into a more efficient and coordinated motor pattern for changing direction.

### Long-term effects on athletic performance

The analysis of long-term interventions first confirms the principle of training specificity. HT training led to a moderate and significant increase in its own 1RM strength (ES = 0.53), yet this strength gain did not effectively transfer to maximal SQ strength, where no significant effect was observed (ES = −0.21). This finding was robust; a sensitivity analysis removing a key outlier study confirmed this lack of transfer, reducing the pooled effect to a clear null (ES = −0.02) and eliminating all heterogeneity. This phenomenon is highly consistent with the findings of Plotkin et al. (2023), who discovered that although HT and SQ training induced similar gluteal hypertrophy, strength gains were strictly specific to the trained movement pattern. This strongly suggests that even similar morphological adaptations (muscle growth) cannot guarantee complete functional transfer between exercises with different neuromuscular coordination patterns. Furthermore, this limited transfer may also be explained by the principle of regional hypertrophy, as HT and SQ likely develop different anatomical regions of the gluteal muscles (Burke et al., 2024). The SQ, as a multi-joint, vertically-loaded exercise, demands significant quadriceps involvement and a distinct pattern of hip extensor activation throughout its range of motion (Araújo et al., 2023; Krause Neto et al., 2020), which is a key factor limiting the transfer of strength. Although this meta-analysis did not directly measure muscle hypertrophy, the research by Kassiano et al. (2024), which showed that adding the HT to a training program more effectively increases gluteus maximus thickness, provides a physiological mechanism supporting the observation of significant HT strength growth.

Regarding the transfer to dynamic athletic performance, this study found that long-term HT training produced small but significant positive effects on linear acceleration sprint (ES = 0.31) and COD speed (ES = 0.25). However, the interpretation of the sprint finding requires extreme caution and methodological scrutiny. A key methodological issue identified in the literature, and a central point of this review, is that some studies test the HT in isolation while others embed it within comprehensive programs. To address this, our sensitivity analysis excluded studies using these combined training protocols. This analysis revealed that the isolated, standalone effect of HT training on sprint performance was substantially smaller (ES = 0.22) and only borderline significant (*p* = 0.05). This strongly suggests that the modest effect observed in the main analysis (ES = 0.31) is likely inflated by, and partially attributable to, the concurrent performance-specific drills (*e.g.*, sprinting) included in those protocols. The isolated effect (ES = 0.22) warrants careful interpretation, especially when contextualized against other training modalities. For instance, a meta-analysis on squat-based strength training reported a strong positive transfer to sprint performance (Seitz et al., 2014), while meta-analyses on plyometric training have shown moderate to large effects (De Villarreal et al., 2012). This comparison suggests that the “force-vector theory” (Loturco et al., 2018), while important, does not fully explain the complex nature of sprint performance. Although the HT, with its anteroposterior resistance vector, directly trains the horizontal force production capacity (Loturco et al., 2018; Williams et al., 2021), our findings suggest this specific transfer (ES = 0.31), and particularly its smaller isolated effect (ES = 0.22), is less potent than the transfer from foundational, vertically-oriented exercises like the squat. This indicates that sprint acceleration, while a horizontal task, may be equally or perhaps more dependent on the foundational capacity to produce massive vertical ground reaction forces—a quality strongly developed by squats (Seitz et al., 2014). Powerful hip extension, even in a vertical orientation, is the primary driver of initial propulsion (Le et al., 2024). Therefore, our data position the HT not as a superior modality for sprinting, but as a complementary tool that specifically targets horizontal force production, which may be underdeveloped by traditional vertical training alone. However, the smaller effect size for COD speed compared to linear sprint reflects the multi-planar, technique-dependent nature of change of direction tasks (Gadea-Uribarri et al., 2024). While the HT develops sagittal-plane hip extension strength effectively, COD performance also requires frontal and transverse plane hip control, deceleration capacity, and sport-specific cutting technique—qualities not directly trained by the HT. Future research should examine whether combining HT with lateral/rotational strength exercises enhances COD transfer.

Critically, our moderator analysis revealed that the benefits for acceleration were significantly greater in younger athletes (<18 years, ES = 0.50), suggesting that this population may be more responsive to horizontally-oriented training stimuli or that their sprinting technique is less developed, allowing more room for improvement from foundational strength gains (Gillen et al., 2024; Pote et al., 2024). The enhanced responsiveness of adolescent athletes to HT training for sprint acceleration may reflect several developmental factors. During adolescence, the neuromuscular system exhibits heightened plasticity, and horizontal force production capacities are still developing compared to mature athletes. Furthermore, younger athletes typically have less training history with horizontal-loading exercises, providing greater stimulus novelty (Gillen et al., 2024; Villanueva-Guerrero et al., 2023).

In stark contrast, and further reinforcing the force-vector theory, long-term HT training showed no statistically significant improvement in jumping performance (ES = 0.14, *p* = 0.11), indicating no clinically meaningful transfer. While the sample size provided robustness (*I*^2^ = 0.00%, Egger’s test *p* = 0.84), the statistical power was insufficient to definitively rule out the existence of very small, non-clinical effects, thus requiring us to interpret the ES = 0.14 finding as no meaningful improvement in jump performance. The lack of improvement in the vertically-oriented CMJ is straightforwardly explained by this principle. The non-significant result for the SLJ, a horizontal task, is more nuanced. An optimal SLJ requires not only horizontal propulsion but also a significant vertical component to maximize flight time and achieve an ideal launch angle, involving a coordinated triple extension of the hip, knee, and ankle joints (Lees, Vanrenterghem & De Clercq, 2004; Robertson & Fleming, 1987). The HT, being a hip-dominant movement, may not sufficiently train the other components or the intricate inter-muscular coordination required for this multi-faceted skill (Brazil et al., 2021). Therefore, while the HT develops a key component of the SLJ (hip extension), its specificity is too narrow to significantly enhance the entire, multi-faceted skill. This highlights that for complex skills, the transfer from a general strength exercise, even one with a similar force vector, may be limited without more direct practice of the skill itself.

### Practical applications

The findings of this meta-analysis offer clear, evidence-based recommendations. For acute performance enhancement, the HT can be effectively used to improve sprint acceleration. To optimize the PAPE effect, practitioners should implement multiple sets and ensure a recovery interval of ≥4 min is provided between the HT and the subsequent sprint. In long-term programming, the HT is a primary tool for increasing exercise-specific hip extension strength and developing horizontal force production. Its independent contribution to sprint acceleration appears to be very small and of low certainty. However, it serves as a valuable complementary tool, which, when integrated within a comprehensive program that includes performance-specific drills, translates to small but significant improvements in initial sprint acceleration.

### Limitations

Several limitations must be acknowledged. First, while this review was registered on OSF, registration on PROSPERO, the preferred platform for intervention systematic reviews, was not completed prior to study commencement. Second, we acknowledge that our search strategy, while comprehensive across published databases, may have limited capture of unpublished data and trial registries, potentially contributing to publication bias as evidenced by the significant Egger’s test (*p* = 0.001) for acute sprint performance. Third, a key limitation stems from the methodological quality of the primary literature. As our PEDro and RoB 2 assessments revealed, weaknesses including small sample sizes and a near-universal lack of blinding (of subjects, therapists, and assessors) and allocation concealment introduce a high risk of bias. This lack of blinding is a critical issue, as performance bias (*e.g.*, greater encouragement from coaches) and detection bias (*e.g.*, biased assessment of outcomes) likely favor the intervention groups. In addition to this, a formal assessment of selective outcome reporting was not feasible, as the majority of included primary studies lacked necessary prospective registration data required for comparison. Consequently, it is probable that the ‘small’ to ‘moderate’ positive effects observed in our analysis (*e.g.*, for sprinting, ES = 0.31) represent an overestimation of the true, independent effect of the HT. This high risk of bias is the primary reason our GRADE assessment rated the certainty of evidence as ‘Low’ or ‘Very Low’ for most performance outcomes. Fourth, a design limitation within the primary studies is the use of confounding variables. Some included studies used the HT as the foundational strength exercise but also incorporated other auxiliary exercises. As discussed, this makes it difficult to attribute the observed effects entirely to the HT alone, as evidenced by our sensitivity analysis for sprint performance. Fifth, significant heterogeneity was observed in the acute sprint analysis (*I*^2^ = 81.50%). Although moderator analysis identified recovery duration and volume as significant sources of this variance, substantial heterogeneity persisted even within subgroups. This suggests that while PAPE is achievable, its optimal prescription remains complex. sixth, a limitation related to the primary studies is the general absence of reported test-retest reliability for their performance measures. The lack of reported reliability introduces potential ’noise’ or measurement error from the original data. This underlying variance not only reduces the overall precision of our pooled effect size estimates (*i.e.,* widens their confidence intervals) but may also be a contributing factor to the high heterogeneity observed in some analyses. Finally, the conclusions should be interpreted with caution due to the limited number of primary studies available for certain moderator analyses (*e.g.*, *k* = 2 or *k* = 3 for some control-type subgroups), which limited the statistical power and certainty of these specific subgroup findings.

### Future directions

To deepen the understanding of the HT’s effects and address current research gaps, future investigations should advance in several key directions. First, while the ‘force-vector theory’ provides a robust explanatory framework, future studies should aim to explore its nuances with greater precision. On a performance level, this involves moving beyond validating the HT in isolation and conducting direct comparative studies to determine its efficacy against other horizontally-oriented exercises, such as sled pushes or kettlebell swings, for enhancing specific athletic tasks. Mechanistically, research could then aim to explain these performance outcomes by, for instance, using kinetic analysis to directly measure how different training modalities alter horizontal ground reaction forces during sprinting, or employing advanced imaging to map how specific patterns of regional muscle hypertrophy correlate with functional transfer. Second, there is a clear need to enhance the methodological quality and specificity of intervention studies. Many studies in this review combined HT with other auxiliary exercises; future randomized controlled trials should systematically compare isolated HT training against combined protocols to clarify the unique contribution of the HT itself. These trials must also prioritize larger sample sizes and proper allocation concealment to increase statistical power and minimize the risk of bias, thereby increasing the certainty of the evidence. Third, research should focus on optimizing training prescription and integration. To develop more precise and individualized PAPE protocols, future studies must clarify the interplay between conditioning load, volume, and athlete-specific factors (*e.g.*, strength level), moving beyond group-based recommendations. Furthermore, longitudinal research is needed to investigate the optimal integration of HT with traditional vertical exercises within long-term, periodized plans, comparing the effects of concurrent *versus* block-periodization models on athletic development, particularly in youth populations where the benefits appear most pronounced. Finally, the scope of inquiry must be broadened to enhance external validity and generalizability. This includes intensifying research specifically on female athletes to address the current gender imbalance. A critical step would be to conduct sex-stratified randomized controlled trials directly comparing volume-equated HT and traditional vertical exercises (*e.g.*, squat), to explore how sex may moderate the HT’s role in performance and injury-risk mitigation given their unique anatomical and physiological characteristics. Additionally, future trials should expand the scope of performance outcomes beyond the sagittal plane, assessing the transfer of HT training to the multi-planar and rotational tasks that are critical to a wider range of sports. By incorporating more varied populations and assessing a wider array of relevant performance outcomes, the true applicability of the HT in diverse athletic contexts can be more definitively established.

## Conclusions

This meta-analysis establishes the HT not as a universally superior training modality, but as a specialized tool with highly specific applications. Acutely, it serves as an effective conditioning tool for PAPE, moderately improving subsequent sprint performance; however, this benefit is highly dependent on specific protocols, namely the use of multiple sets and an adequate recovery period (≥4 min). Long-term, HT training reliably develops exercise-specific strength and provides small, vector-specific transfer to linear acceleration and COD speed, though these benefits fail to transfer to squat strength or jumping performance. Critically, the overall certainty of this evidence is ‘Low’ to ‘Very Low’, and our sensitivity analysis revealed that the small sprint transfer was reduced to a borderline-significant effect when studies using combined-training protocols were excluded. Therefore, we conclude that the HT is an effective tool for developing its own specific strength and should be considered a valuable complement to, rather than a standalone replacement for, a complete athletic development program that includes traditional, vertically-oriented exercises.

## Supplemental Information

10.7717/peerj.20785/supp-1 Supplemental Information 1 The complete and database-specific Search strategy

10.7717/peerj.20785/supp-2 Supplemental Information 2 Sensitivity analysis results * Note* NA, no studies were excluded; *k*, number of comparisons; ES, Effect sizes (Hedges’ g); CI, Confidence Interval; a , Analysis performed after excluding studies identified as potential outliers due to large deviations; b , Analysis performed after excluding studies that utilized combined training protocols (i.e., HT + auxiliary exercises).

10.7717/peerj.20785/supp-3 Supplemental Information 3 Recommendation, Assessment, Development and Evaluation tool for the assessment of certainty of evidence * Note* CI: Confidence Interval; ES: Effect sizes (Hedges’ g); *k*, Number of comparisons; *n*, Number of participants. ^1^ Downgraded by one level due to high risk of bias (majority of studies rated “some concerns” or “high risk” on RoB 2 tool). ^2^ Downgraded by one level due to serious or very serious unexplained heterogeneity (*I*^2^ ¿50%). ^3^ Downgraded by one level due to serious indirectness. The primary studies for this outcome used combined training protocols (HT + auxiliary exercises). A sensitivity analysis (Table S2) confirmed this indirectness impacted the robustness of the effect size (reducing the pooled ES to 0.22 with *p* = 0.05). ^4^ Downgraded by one level due to serious imprecision (95% CI crosses the line of no effect). ^5^ Downgraded by one level due to serious imprecision (low number of studies (*k* < 5) and total participants (*n* < 100)). ^6^ Downgraded by one level due to serious publication bias (Egger’s test *p* < 0.05). ^a^ Heterogeneity for ‘Back squat strength’ (I 2 = 75.04%) was considered explained, as a sensitivity analysis (excluding Barbalho et al., 2020) reduced heterogeneity to 0.00%; therefore, this domain was not downgraded. ^*b*^ Indirectness due to combined training protocols (HT + auxiliary exercises) was assessed. A sensitivity analysis excluding these studies (Bartolomei et al., 2024); Sanchez-Sabate et al.2024) revealed no significant impact on the pooled effect size; therefore, this domain was not downgraded.

10.7717/peerj.20785/supp-4 Supplemental Information 4 Moderator analysis * Note* ES, effect size; CI, confidence intervals; *I*^2^,heterogeneity; *p*-diff, *p* for Subgroup Differences; *,indicate statistical significance (*p* ¡ 0.05); HT, hip thrust; SQ, squat; DL, deadlift; CG, control group; Mix, both male and female.

10.7717/peerj.20785/supp-5 Supplemental Information 5 Measurement Device Specifications

10.7717/peerj.20785/supp-6 Supplemental Information 6 Contour-enhanced funnel plots

10.7717/peerj.20785/supp-7 Supplemental Information 7 PRISMA checklist

10.7717/peerj.20785/supp-8 Supplemental Information 8 Meta-Analysis Raw Data The data comprises the original measurements extracted from the cited literature, including sample sizes, means, standard deviations for both the experimental and control groups, as well as effect sizes (Cohen’s d), standard errors (SE), and 95% confidence intervals (CI) where applicable.

## Additional Information and Declarations

### Data Availability

The following information was supplied regarding data availability: The raw measurements are available in the Supplemental File.
