# Supplementary material for: Acute and long-term effects of hip thrust training on athletic performance: a systematic review and meta-analysis
Source: PeerJ. 2026 Feb 27;14:e20785. doi: 10.7717/peerj.20785 (PMC12951884; doi:10.7717/peerj.20785)
Supplement: Supplemental Information 1 [file peerj-14-20785-s001.docx]

**Title: Acute and Long-Term Effects of Hip Thrust Training on Athletic Performance: A Systematic Review and Meta-Analysis**

**Journal Name: *PeerJ***

**Authors:** Shengfa Lin^1^,Mengna Chen^1^,Xiaolan Yi^1^, Yuhao Li^1^, Ruidong Liu^1, 2,^*

**Affiliations:**

^1^ Sports Coaching College, Beijing Sport University, Haidian District, Beijing, China

^2^ Key Laboratory of Sport Training of General Administration of Sport of China, Beijing Sport University, Haidian District, Beijing, China

Corresponding Author:

Ruidong Liu

48 Xinxi Road, Haidian District, Beijing, 100084, China

Email address: lrd5156@bsu.edu.cn

**Table S1:** **The complete and database-specific Search strategy.**

| **Database** | **Search strategy** | **Results** |
| --- | --- | --- |
| PubMed | ("hip thrust"[Title/Abstract] OR "barbell hip thrust"[Title/Abstract] OR "glute* bridge"[Title/Abstract] OR "hip bridge"[Title/Abstract] OR "hip extensor exercise"[Title/Abstract] OR "hip extension exercise"[Title/Abstract]) AND ("performance"[Title/Abstract] OR "strength"[Title/Abstract] OR "power"[Title/Abstract] OR "speed"[Title/Abstract] OR "sprint"[Title/Abstract] OR "change of direction"[Title/Abstract] OR "COD"[Title/Abstract] OR "rate of force development"[Title/Abstract] OR "RFD"[Title/Abstract] OR "vertical jump"[Title/Abstract] OR "VJ"[Title/Abstract] OR "Countermovement Jump"[Title/Abstract] OR "CMJ"[Title/Abstract] OR "agility"[Title/Abstract] OR "1RM"[Title/Abstract] OR "acute"[Title/Abstract] OR "post activation"[Title/Abstract] OR "conditioning activity"[Title/Abstract] OR "post-activation potentiation"[Title/Abstract] OR "PAPE"[Title/Abstract] OR "PAP"[Title/Abstract] OR "EMG"[Title/Abstract] OR "muscle activation"[Title/Abstract] OR "electromyograph*"[Title/Abstract] OR "muscle thickness"[Title/Abstract] OR "cross-sectional area"[Title/Abstract] OR "hypertroph*"[Title/Abstract] OR "muscle growth"[Title/Abstract]) | 106 |
| Scopus | TITLE-ABS-KEY ( ( "hip thrust" OR "barbell hip thrust" OR "glute* bridge" OR "hip bridge" OR "hip extensor exercise" OR "hip extension exercise" ) AND ( "performance" OR "strength" OR "power" OR "speed" OR "sprint" OR "change of direction" OR "COD" OR "rate of force development" OR "RFD" OR "vertical jump" OR "VJ" OR "Countermovement Jump" OR "CMJ" OR "agility" OR "1RM" OR "acute" OR "post activation" OR "conditioning activity" OR "post-activation potentiation" OR "PAPE" OR "PAP" OR "EMG" OR "muscle activation" OR "electromyograph*" OR "muscle thickness" OR "cross-sectional area" OR "hypertroph*" OR "muscle growth" ) ) | 152 |
| CINHAL Complete | (“hip thrust” OR “barbell hip thrust” OR “glute* bridge” OR “hip bridge” OR “hip extensor exercise” OR “hip extension exercise”) AND (“performance” OR “strength” OR “power” OR “speed” OR “sprint” OR “change of direction” OR “COD” OR “rate of force development” OR “RFD” OR “vertical jump” OR “VJ” OR “Countermovement Jump” OR “CMJ” OR “agility” OR “1RM” OR “acute” OR “post activation” OR “conditioning activity” OR “post-activation potentiation” OR “PAPE” OR “PAP” OR “EMG” OR “muscle activation” OR “electromyograph*” OR “muscle thickness” OR “cross-sectional area” OR “hypertroph*” OR “muscle growth”) | 59 |
| MELINE Complete | (“hip thrust” OR “barbell hip thrust” OR “glute* bridge” OR “hip bridge” OR “hip extensor exercise” OR “hip extension exercise”) AND (“performance” OR “strength” OR “power” OR “speed” OR “sprint” OR “change of direction” OR “COD” OR “rate of force development” OR “RFD” OR “vertical jump” OR “VJ” OR “Countermovement Jump” OR “CMJ” OR “agility” OR “1RM” OR “acute” OR “post activation” OR “conditioning activity” OR “post-activation potentiation” OR “PAPE” OR “PAP” OR “EMG” OR “muscle activation” OR “electromyograph*” OR “muscle thickness” OR “cross-sectional area” OR “hypertroph*” OR “muscle growth”) | 108 |
| SPORTDiscus | (“hip thrust” OR “barbell hip thrust” OR “glute* bridge” OR “hip bridge” OR “hip extensor exercise” OR “hip extension exercise”) AND (“performance” OR “strength” OR “power” OR “speed” OR “sprint” OR “change of direction” OR “COD” OR “rate of force development” OR “RFD” OR “vertical jump” OR “VJ” OR “Countermovement Jump” OR “CMJ” OR “agility” OR “1RM” OR “acute” OR “post activation” OR “conditioning activity” OR “post-activation potentiation” OR “PAPE” OR “PAP” OR “EMG” OR “muscle activation” OR “electromyograph*” OR “muscle thickness” OR “cross-sectional area” OR “hypertroph*” OR “muscle growth”) | 106 |
