# Supplementary material for: Acute and long-term effects of hip thrust training on athletic performance: a systematic review and meta-analysis
Source: PeerJ. 2026 Feb 27;14:e20785. doi: 10.7717/peerj.20785 (PMC12951884; doi:10.7717/peerj.20785)
Supplement: Supplemental Information 2 — Note NA, no studies were excluded; k, number of comparisons; ES, Effect sizes (Hedges’ g); CI, Confidence Interval; a , Analysis performed after excluding studies identified as potential outliers due to large deviations; b , Analysis performed after excluding studies that utilized combined training protocols (i.e., HT + auxiliary exercises). [file peerj-14-20785-s002.docx]

**Title: Acute and Long-Term Effects of Hip Thrust Training on Athletic Performance: A Systematic Review and Meta-Analysis**

**Journal Name: *PeerJ***

**Authors:** Shengfa Lin^1^,Mengna Chen^1^,Xiaolan Yi^1^, Yuhao Li^1^, Ruidong Liu^1, 2,^*

**Affiliations:**

^1^ Sports Coaching College, Beijing Sport University, Haidian District, Beijing, China

^2^ Key Laboratory of Sport Training of General Administration of Sport of China, Beijing Sport University, Haidian District, Beijing, China

Corresponding Author:

Ruidong Liu

48 Xinxi Road, Haidian District, Beijing, 100084, China

Email address: lrd5156@bsu.edu.cn

**Table S2 Sensitivity analysis results.**

| Outcomes | | Dimension of the analysis | *k* | ES | 95%CI | *p* | Heterogeneity |
| --- | --- | --- | --- | --- | --- | --- | --- |
| Acute effect | Linear acceleration sprint performance | NA | 46 | 0.55 | 0.31, 0.78 | *p* < 0.001 | 81.50 |
|  |  | Dello et al., 2017^a^ | 35 | 0.51 | 0.23, 0.80 | *p* < 0.001 | 83.31 |
|  |  | Dello et al., 2018^a^ | 27 | 0.40 | 0.21, 0.59 | *p* < 0.001 | 49.83 |
| Chronic effect | Hip thrust strength | NA | 12 | 0.53 | 0.26, 0.81 | *p* < 0.001 | 0.00 |
|  |  | Bartolomei et al., 2024^b^ | 11 | 0.53 | 0.24, 0.82 | *p* < 0.001 | 0.00 |
|  | Back squat strength | NA | 11 | -0.21 | -0.65, 0.24 | *p* = 0.37 | 55.49 |
|  |  | Bartolomei et al., 2024^b^ | 10 | -0.20 | -0.70, 0.30 | *p* = 0.43 | 59.85 |
|  |  | Barbalho et al. 2020^a^ | 10 | -0.02 | -0.32, 0.28 | *p* = 0.90 | 0.00 |
|  | Linear acceleration sprint performance | NA | 24 | 0.31 | 0.12, 0.51 | *p* < 0.001 | 0.00 |
|  |  | Bartolomei et al., 2024; Sanchez-Sabate et al.2024^b^ | 20 | 0.22 | 0.00, 0.43 | *p* = 0.05 | 0.00 |
|  | Change of direction | NA | 7 | 0.25 | 0.02, 0.48 | *p* = 0.03 | 0.00 |
|  |  | Sanchez-Sabate et al.2024^b^ | 6 | 0.25 | 0.02, 0.49 | *p* = 0.04 | 0.00 |
|  | Jump performance | NA | 25 | 0.14 | -0.03, 0.30 | *p* = 0.11 | 0.00 |
|  |  | Bartolomei et al., 2024; Sanchez-Sabate et al.2024^b^ | 22 | 0.14 | -0.03, 0.31 | *p* = 0.13 | 0.00 |

*Note* NA, no studies were excluded; *k*, number of comparisons; ES, Effect sizes (Hedges' g); CI, Confidence Interval; ^a^, Analysis performed after excluding studies identified as potential outliers due to large deviations; ^b^, Analysis performed after excluding studies that utilized combined training protocols (i.e., HT + auxiliary exercises).
