# Supplementary material for: Acute and long-term effects of hip thrust training on athletic performance: a systematic review and meta-analysis
Source: PeerJ. 2026 Feb 27;14:e20785. doi: 10.7717/peerj.20785 (PMC12951884; doi:10.7717/peerj.20785)
Supplement: Supplemental Information 3 — Note CI: Confidence Interval; ES: Effect sizes (Hedges’ g); k, Number of comparisons; n, Number of participants. 1 Downgraded by one level due to high risk of bias (majority of studies rated “some concerns” or “high risk” on RoB 2 tool). 2 Downgraded by one level due to serious or very serious unexplained heterogeneity (I2 ¿50%). 3 Downgraded by one level due to serious indirectness. The primary studies for this outcome used combined training protocols (HT + auxiliary exercises). A sensitivity analysis (Table S2) confirmed this indirectness impacted the robustness of the effect size (reducing the pooled ES to 0.22 with p = 0.05). 4 Downgraded by one level due to serious imprecision (95% CI crosses the line of no effect). 5 Downgraded by one level due to serious imprecision (low number of studies (k < 5) and total participants (n < 100)). 6 Downgraded by one level due to serious publication bias (Egger’s test p < 0.05). a Heterogeneity for ‘Back squat strength’ (I 2 = 75.04%) was considered explained, as a sensitivity analysis (excluding Barbalho et al., 2020) reduced heterogeneity to 0.00%; therefore, this domain was not downgraded. b Indirectness due to combined training protocols (HT + auxiliary exercises) was assessed. A sensitivity analysis excluding these studies (Bartolomei et al., 2024); Sanchez-Sabate et al.2024) revealed no significant impact on the pooled effect size; therefore, this domain was not downgraded. [file peerj-14-20785-s003.docx]

**Title: Acute and Long-Term Effects of Hip Thrust Training on Athletic Performance: A Systematic Review and Meta-Analysis**

**Journal Name: *PeerJ***

**Authors:** Shengfa Lin^1^,Mengna Chen^1^,Xiaolan Yi^1^, Yuhao Li^1^, Ruidong Liu^1, 2,^*

**Affiliations:**

^1^ Sports Coaching College, Beijing Sport University, Haidian District, Beijing, China

^2^ Key Laboratory of Sport Training of General Administration of Sport of China, Beijing Sport University, Haidian District, Beijing, China

Corresponding Author:

Ruidong Liu

48 Xinxi Road, Haidian District, Beijing, 100084, China

Email address: lrd5156@bsu.edu.cn

**Table S3 Recommendation, Assessment, Development and Evaluation tool for the assessment of certainty of evidence.**

| **Outcome** | | ***k*** | ***n*** | **Certainty of the Evidence (GRADE)** | **Risk of Bias** | **Inconsistency** | **Indirectness** | **Imprecision** | **Publication bias** | **ES**  **(95% CI)** |
| --- | --- | --- | --- | --- | --- | --- | --- | --- | --- | --- |
| Acute effect | Linear acceleration sprint performance | 46 | 102 | ⊕◯◯◯  very low | Serious¹ | Serious² | Not serious | Not serious | Serious^6^ | 0.55  (0.31, 0.78) |
|  | Jump performance | 5 | 29 | ⊕⊕◯◯  low | Serious¹ | Not serious | Not serious | Serious^4, 5^ | Undetected | 0.18  (-0.16, 0.52) |
| Chronic effect | Hip thrust strength | 12 | 229 | ⊕⊕⊕◯  Moderate | Serious¹ | Not serious | Not serious^b^ | Not serious | Undetected | 0.53  (0.26, 0.81) |
|  | Back squat strength | 11 | 208 | ⊕⊕◯◯  low | Serious¹ | Not serious^a^ | Not serious^b^ | Serious^4^ | Undetected | -0.21  (-0.65, 0.24) |
|  | Linear acceleration sprint performance | 24 | 192 | ⊕⊕◯◯  low | Serious¹ | Not serious | Serious^3^ | Not serious | Undetected | 0.31  (0.12, 0.51) |
|  | Change of direction | 7 | 56 | ⊕⊕◯◯  low | Serious¹ | Not serious | Not serious^b^ | Serious^5^ | Undetected | 0.25  (0.02, 0.48) |
|  | Jump performance | 25 | 186 | ⊕⊕◯◯  low | Serious¹ | Not serious | Not serious^b^ | Serious^4^ | Undetected | 0.14  (-0.03, 0.30) |

*Note* CI: Confidence Interval; ES: Effect sizes (Hedges' g); *k*, Number of comparisons; *n*, Number of participants.

¹ Downgraded by one level due to high risk of bias (majority of studies rated “some concerns” or “high risk” on RoB 2 tool).

^2^ Downgraded by one level due to serious or very serious unexplained heterogeneity (I² > 50%).

^3^ Downgraded by one level due to serious indirectness. The primary studies for this outcome used combined training protocols (HT + auxiliary exercises). A sensitivity analysis (Table S2) confirmed this indirectness impacted the robustness of the effect size (reducing the pooled ES to 0.22 with *p* = 0.05).

^4^ Downgraded by one level due to serious imprecision (95% CI crosses the line of no effect).

^5^ Downgraded by one level due to serious imprecision (low number of studies (*k* < 5) and total participants (*n* < 100)).

^6^ Downgraded by one level due to serious publication bias (Egger's test *p* < 0.05).

^a^ Heterogeneity for ‘Back squat strength’ (I^2^ = 75.04%) was considered explained, as a sensitivity analysis (excluding Barbalho et al., 2020) reduced heterogeneity to 0.00%; therefore, this domain was not downgraded.

^b^ Indirectness due to combined training protocols (HT + auxiliary exercises) was assessed. A sensitivity analysis excluding these studies (Bartolomei et al., 2024; Sanchez-Sabate et al.2024) revealed no significant impact on the pooled effect size; therefore, this domain was not downgraded.
