# Supplementary material for: Acute and long-term effects of hip thrust training on athletic performance: a systematic review and meta-analysis
Source: PeerJ. 2026 Feb 27;14:e20785. doi: 10.7717/peerj.20785 (PMC12951884; doi:10.7717/peerj.20785)
Supplement: Supplemental Information 4 — Note ES, effect size; CI, confidence intervals; I2,heterogeneity; p-diff, p for Subgroup Differences; *,indicate statistical significance (p ¡ 0.05); HT, hip thrust; SQ, squat; DL, deadlift; CG, control group; Mix, both male and female. [file peerj-14-20785-s004.docx]

**Title: Acute and Long-Term Effects of Hip Thrust Training on Athletic Performance: A Systematic Review and Meta-Analysis**

**Journal Name: *PeerJ***

**Authors:** Shengfa Lin^1^,Mengna Chen^1^,Xiaolan Yi^1^, Yuhao Li^1^, Ruidong Liu^1, 2,^*

**Affiliations:**

^1^ Sports Coaching College, Beijing Sport University, Haidian District, Beijing, China

^2^ Key Laboratory of Sport Training of General Administration of Sport of China, Beijing Sport University, Haidian District, Beijing, China

Corresponding Author:

Ruidong Liu

48 Xinxi Road, Haidian District, Beijing, 100084, China

Email address: lrd5156@bsu.edu.cn

**Table S4: Moderator analysis.**

| Outcome | Moderator | Number of comparisons | ES (Hedges’g) | 95%CI | I^2^ (%) | *p*-diff |
| --- | --- | --- | --- | --- | --- | --- |
| Acute effect |  |  |  |  |  |  |
| Linear acceleration sprint | Recovery | Immediate (< 4min) = 10 | -0.44 | (-0.78, -0.11) | 62.02 | < 0.001* |
|  |  | short (4 - 7 min) = 22 | 0.69 | (0.47, 0.91) | 55.48 |  |
|  |  | moderate (8 - 10 min) = 14 | 1.05 | (0.64, 1.47) | 79.49 |  |
|  | Intensity | ≥ 85%1RM = 25 | 0.38 | (0.07, 0.70) | 81.03 | 0.13 |
|  |  | < 85%1RM = 21 | 0.74 | (0.39, 1.10) | 81.60 |  |
|  | Age | ≥ 18 years = 34 | 0.64 | (0.33, 0.95) | 85.94 | 0.06 |
|  |  | < 18 years = 12 | 0.28 | (0.08, 0.49) | 0.00 |  |
|  | Volume | Single sets = 14 | 0.25 | (0.07, 0.44) | 0.00 | 0.03* |
|  |  | Multiple sets = 32 | 0.67 | (0.34, 1.00) | 86.48 |  |
|  | Risk of bias | High risk = 6 | 0.22 | (-0.04, 0.48) | 0.00 | 0.05 |
|  |  | Some concerns = 40 | 0.60 | (0.33, 0.87) | 83.55 |  |
| Long-term training adaptations |  |  |  |  |  |  |
| HT strength | Control types | HT vs. SQ = 7 | 0.50 | (0.16, 0.84) | 0.00 | 0.52 |
|  |  | HT vs. DL = 2 | 0.23 | (-0.57, 1.02) | 0.00 |  |
|  |  | HT vs. CG = 3 | 0.77 | (0.21, 1.33) | 0.00 |  |
|  | Age | ≥ 18 years = 10 | 0.45 | (0.15, 0.76) | 0.00 | 0.23 |
|  |  | < 18 years = 2 | 0.89 | (0.24, 1.54) | 0.00 |  |
|  | Sex | Male = 5 | 0.50 | (0.04, 0.97) | 25.94 | 0.75 |
|  |  | Female = 2 | 0.68 | (0.04, 1.33) | 0.00 |  |
|  |  | Mix = 5 | 0.49 | (0.03, 0.96) | 0.00 |  |
|  | Sport | Athlete = 6 | 0.76 | (0.33, 1.18) | 0.00 | 0.18 |
|  |  | Non-athlete = 6 | 0.37 | (0.01, 0.73) | 0.00 |  |
|  | Experience | Trained = 8 | 0.61 | (0.29, 0.93) | 0.00 | 0.35 |
|  |  | Untrained = 3 | 0.32 | (-0.22,0.85) | 0.00 |  |
|  | Frequency | ≥ 3 week^-1^ = 5 | 0.63 | (0.15, 1.10) | 0.00 | 0.63 |
|  |  | < 3 week-1 = 7 | 0.48 | (0.15, 0.82) | 0.00 |  |
|  | Duration | ≥ 8week = 3 | 0.78 | (0.28, 1.27) | 0.00 | 0.25 |
|  |  | < 8week = 9 | 0.43 | (0.10, 0.75) | 0.00 |  |
|  | Risk of bias | High risk = 8 | 0.56 | (0.21, 0.91) | 0.00 | 0.78 |
|  |  | Some concerns = 4 | 0.48 | (0.04, 0.92) | 0.00 |  |
| SQ strength | Control types | HT vs. SQ = 7 | -0.44 | (-1.01, 0.12) | 60.16 | 0.15 |
|  |  | HT vs. DL = 2 | 0.00 | (-0.79, 0.79) | 0.00 |  |
|  |  | HT vs. CG = 2 | 0.56 | (-0.28, 1.40) | 21.79 |  |
|  | Age | ≥ 18 years = 9 | -0.24 | (-0.79, 0.32) | 63.39 | 0.82 |
|  |  | < 18 years = 2 | -0.14 | (-0.75, 0.47) | 0.00 |  |
|  | Sex | Male = 5 | 0.04 | (-0.41, 0.16) | 20.74 | 0.61 |
|  |  | Female = 2 | -1.10 | (-3.59, 1.40) | 91.24 |  |
|  |  | Mix = 4 | -0.16 | (-0.71, 0.38) | 0.00 |  |
|  | Sport | Athlete = 6 | 0.10 | (-0.31, 0.51) | 0.00 | 0.16 |
|  |  | Non-athlete = 5 | -0.55 | (-1.34, 0.24) | 71.55 |  |
|  | Experience | Trained = 8 | -0.27 | (-0.90, 0.36) | 67.89 | 0.69 |
|  |  | Untrained = 3 | -0.10 | (-0.63, 0.43) | 55.49 |  |
|  | Frequency | ≥ 3 week^-1^ = 5 | 0.12 | (-0.36, 0.60) | 4.99 | 0.18 |
|  |  | < 3 week^-1^ = 6 | -0.44 | (-1.10, 0.23) | 67.53 |  |
|  | Duration | ≥ 8week = 2 | -0.73 | (-3.93, 2.47) | 95.16 | 0.72 |
|  |  | < 8week = 9 | -0.14 | (-0.46, 0.18) | 0.00 |  |
|  | Risk of bias | High risk = 8 | 0.01 | (-0.34, 0.35) | 0.00 | 0.26 |
|  |  | Some concerns = 3 | -0.83 | (-2.24, 0.59) | 84.07 |  |
| Linear acceleration sprint | Control types | HT vs. SQ = 13 | 0.46 | (0.20, 0.72) | 0.00 | 0.22 |
|  |  | HT vs. DL = 2 | -0.02 | (-0.90, 0.82) | 0.00 |  |
|  |  | HT vs. CG = 9 | 0.14 | (-0.18, 0.45) | 0.00 |  |
|  | Age | ≥ 18 years = 10 | -0.02 | (-0.34, 0.30) | 0.00 | 0.01* |
|  |  | < 18 years = 14 | 0.50 | (0.26, 0.74) | 0.00 |  |
|  | Sex | Male = 10 | 0.56 | (0.19, 0.93) | 30.44 | 0.09 |
|  |  | Female = 5 | 0.34 | (-0.03, 0.71) | 0.00 |  |
|  |  | Mix = 9 | 0.00 | (-0.35, 0.35) | 0.00 |  |
|  | Sport | Athlete = 21 | 0.38 | (0.17, 0.60) | 0.00 | 0.09 |
|  |  | Non-athlete = 3 | -0.07 | (-0.55, 0.42) | 0.00 |  |
|  | Experience | Trained = 15 | 0.25 | (-0.02, 0.52) | 13.44 | 0.42 |
|  |  | Untrained = 9 | 0.42 | (0.11, 0.73) | 0.00 |  |
|  | Frequency | ≥ 3 week^-1^ = 8 | 0.05 | (-0.33, 0.44) | 0.00 | 0.12 |
|  |  | < 3 week^-1^ = 16 | 0.41 | (0.17, 0.65) | 13.01 |  |
|  | Duration | ≥ 8week = 10 | 0.47 | (0.03, 0.91) | 49.30 | 0.39 |
|  |  | < 8week = 14 | 0.25 | (0.00,0.50) | 0.00 |  |
|  | Risk of bias | High risk = 10 | 0.13 | (-0.19, 0.45) | 0.00 | 0.16 |
|  |  | Some concerns = 14 | 0.44 | (0.15, 0.72) | 24.15 |  |
| Change of direction speed | Control types | HT vs. SQ = 4 | 0.27 | (0.01, 0.54) | 0.00 | 0.73 |
|  |  | HT vs. CG = 2 | 0.18 | (-0.30, 0.65) | 0.00 |  |
|  | Experience | Trained = 4 | 0.09 | (-0.46, 0.65) | 0.00 | 0.55 |
|  |  | Untrained = 3 | 0.28 | (0.03, 0.53) | 0.00 |  |
|  | Risk of bias | High risk = 4 | 0.00 | (-0.58, 0.58) | 0.00 | 0.36 |
|  |  | Some concerns = 3 | 0.29 | (0.04, 0.54) | 0.00 |  |
| Jump performance | Jump types | Countermovement jump = 14 | 0.09 | (-0.11, 0.28) | 0.00 | 0.34 |
|  |  | Standing long jump = 11 | 0.27 | (-0.04, 0.57) | 0.00 |  |
|  | Control types | HT vs. SQ = 14 | 0.03 | (-0.18, 0.25) | 0.00 | 0.38 |
|  |  | HT vs. DL = 4 | 0.13 | (-0.44, 0.70) | 0.00 |  |
|  |  | HT vs. CG = 7 | 0.32 | (-0.02, 0.66) | 11.28 |  |
|  | Age | ≥ 18 years = 14 | 0.09 | (-0.18, 0.37) | 0.00 | 0.70 |
|  |  | < 18 years = 11 | 0.16 | (-0.06, 0.39) | 7.05 |  |
|  | Sex | Male = 7 | 0.09 | (-0.18, 0.36) | 0.00 | 0.87 |
|  |  | Female = 3 | 0.19 | (-0.08, 0.46) | 6.75 |  |
|  |  | Mix = 4 | 0.12 | (-0.26, 0.51) | 0.00 |  |
|  | Sport | Athlete = 19 | 0.14 | (-0.05, 0.32) | 0.00 | 0.98 |
|  |  | Non-athlete = 6 | 0.14 | (-0.26, 0.54) | 0.00 |  |
|  | Experience | Trained = 13 | 0.04 | (-0.22, 0.31) | 0.00 | 0.38 |
|  |  | Untrained = 12 | 0.20 | (-0.02, 0.41) | 0.00 |  |
|  | Frequency | ≥ 3 week^-1^ = 10 | 0.09 | (-0.24, 0.41) | 0.00 | 0.72 |
|  |  | < 3 week^-1^ = 15 | 0.15 | (-0.04, 0.35) | 0.00 |  |
|  | Duration | ≥ 8week = 7 | 0.17 | (-0.23, 0.57) | 11.06 | 0.86 |
|  |  | < 8week = 18 | 0.13 | (-0.05, 0.32) | 0.00 |  |
|  | Risk of bias | High risk = 16 | 0.05 | (-0.20, 0.30) | 0.00 | 0.37 |
|  |  | Some concerns = 9 | 0.22 | (-0.05, 0.50) | 20.98 |  |

*Note* ES, effect size; CI, confidence intervals; I^2^,heterogeneity; *p*-diff, *p* for Subgroup Differences; *,indicate statistical significance (*p*< 0.05); HT, hip thrust; SQ, squat; DL, deadlift; CG, control group; Mix, both male and female.
