# Supplementary material for: Acute and long-term effects of hip thrust training on athletic performance: a systematic review and meta-analysis
Source: PeerJ. 2026 Feb 27;14:e20785. doi: 10.7717/peerj.20785 (PMC12951884; doi:10.7717/peerj.20785)
Supplement: Supplemental Information 5 [file peerj-14-20785-s005.docx]

**Title: Acute and Long-Term Effects of Hip Thrust Training on Athletic Performance: A Systematic Review and Meta-Analysis**

**Journal Name: *PeerJ***

**Authors:** Shengfa Lin^1^,Mengna Chen^1^,Xiaolan Yi^1^, Yuhao Li^1^, Ruidong Liu^1, 2,^*

**Affiliations:**

^1^ Sports Coaching College, Beijing Sport University, Haidian District, Beijing, China

^2^ Key Laboratory of Sport Training of General Administration of Sport of China, Beijing Sport University, Haidian District, Beijing, China

Corresponding Author:

Ruidong Liu

48 Xinxi Road, Haidian District, Beijing, 100084, China

Email address: lrd5156@bsu.edu.cn

**Table S5: Measurement Device Specifications.**

| **Study (Reference)** | **Performance Measure** | **Device Name & Model** |
| --- | --- | --- |
| Çabuk et al. (2025) | Sprint Performance | Not specified (Device not detailed in provided text) |
| Bartolomei et al. (2024) | 1RM Strength (Squat, Hip Thrust) | Barbell (Manufacturer/model not specified) |
|  | Vertical Jump (CMJ) | Photoelectric cells (Optojump, Microgate, Bolzano, Italy) |
|  | Standing Long Jump (SLJ) | Not mentioned specific device, performed on a hard surface |
|  | 20m Sprint | Two pairs of photocells (Witty Gate, Microgate, Bolzano, Italy) |
| Sanchez-Sabate et al. (2024) | Vertical Jump | Optical detection system |
|  | Horizontal Jump (Unilateral HJL​/HJR​) | Measuring tape |
|  | Sprint (T5,T10,T20) | Double-beam photocells (WITTY, MICROGATE) |
|  | Change of Direction (COD, CODL​,CODR​) | Double-beam photocells (WITTY, MICROGATE) |
|  | V-Cut Test (VCUT​) | Double-beam photocells (WITTY, MICROGATE) |
| Plotkin et al. (2023) | Strength (3RM) (Squat, Hip Thrust, Deadlift) | Barbell (Model not specified) |
| Urbański et al. (2023) | Vertical Jump (CMJ) | Contact mat (Chronojump BoscoSystem, Chronojump, Barcelona, Spain) |
| Fernández-Galván et al. (2022) | Sprint Performance (5m, 10m, 20m) | Dual-beam photocells (Witty System, Microgate, Bolzano, Italy) |
| Wilson et al. (2022) | Vertical Jump (CMJ) / Standing Horizontal Jump (SHJ) | Jump mat or force platform (Not specified beyond type) |
|  | Back Squat 1RM / Hip Thrust 1RM | Barbell and plates (Not specified beyond type) |
| Abade et al. (2021) | Vertical Jump (SJ, CMJ) | Optojump device (Microgate, Bolzano, Italy) |
|  | Horizontal Jump (SLJ) | Standard measuring tape |
|  | Sprint Time (10m, 20m) | Infrared timing system (Globus Ergo Timer; Timing System, Codogné, Italy) |
| Atalağ et al. (2020) | Vertical Jump (CMJ) | Contact mat (Chronojump BoscoSystem, Chronojump, Barcelona, Spain) |
|  | Sprint Performance (20 m Sprint) | 4 single-beam photocells (Microgate Witty System, Microgate, Bolzano, Italy) |
| Barbalho et al. (2020) | Muscle Thickness (MT) (Gluteus Maximus, Vastus Lateralis) | B-mode ultrasound (Toshiba Tosbe model, 7.5 MHz linear transduction) |
| Carbone et al. (2020) | Sprint Performance (10m, 20m) | Dual beam photocells (Witty, Microgate, Bolzano, Italy) |
|  | Maximum Strength (1RM Hip Thrust, 1RM Back Squat) | Olympic Barbell and Plates |
| Millar et al. (2020) | Sprint Time (36.6m Dash) | Timing gates (Brower Timing System) |
|  | Pro-Agility Shuttle | Timing gates (Brower Timing System) |
|  | Vertical Jump (VJ) | Vertec apparatus (Sports Imports) |
|  | Broad Jump (BJ) | Tape measure (Lufkin Reel Rewind Tape, 100') |
| Orjalo et al. (2020) | Change of Direction (COD) Speed (Pro Agility Test) | Electronic timing gates (Microgate Witty Timing System, Microgate, Bolzano, Italy) |
|  | PAP Exercise (Barbell Hip Thrust) | Barbell |
| González-García et al. (2019) | Maximum Strength (1RM Back Squat) | Olympic Barbell and plates (Manufacturer/model not specified) |
|  | Maximum Strength (1RM Hip Thrust) | Olympic Barbell and plates, hip thrust bench (Manufacturer/model not specified) |
|  | Speed (10m Sprint, 20m Sprint, 30m Sprint) | Electronic timing gates (Witty System, Microgate, Bolzano, Italy) |
|  | Agility (T-test) | Electronic timing gates (Witty System, Microgate, Bolzano, Italy) |
|  | Vertical Jump (CMJ) | Contact mat (Ergo-Jump, Globus Italia, Codognè, Italy) |
| Hammond et al. (2019) | Maximum Strength (1RM Back Squat) | Olympic Barbell and Plates (Manufacturer/model not specified) |
|  | Maximum Strength (1RM Hip Thrust) | Olympic Barbell and Plates (Manufacturer/model not specified), Hip Thrust bench/apparatus |
| Jarvis et al. (2019) | Sprint Time (40m, Splits) | Infrared timing gates (Brower, Wireless TC Timing System) |
|  | 1RM Hip Thrust | Standardized barbell/plates |
| Wilson et al. (2019) | Maximum Strength (1RM Back Squat) | Olympic Barbell and Plates (Manufacturer/model not specified) |
| Dello et al. (2018) | 10m Sprint, 20m Sprint, 30m Sprint | Two pairs of single-beam photocells (Globus Electronics, Codogne, Italy) |
| Contreras et al. (2017) | Front Squat 1RM | Olympic-style barbell, iron plates, power rack (Equipment not specified beyond type) |
|  | Hip Thrust 1RM | Olympic-style barbell, iron plates, hip thrust bench (Equipment not specified beyond type) |
|  | Vertical Jump (CMJ) | Just Jump System (Probotics, Inc., Huntsville, AL) |
|  | 10m Sprint, 20m Sprint | Electronic timing gates (Dashr timing system, Lincoln, NE) |
| Dello et al. (2017) | 10m Sprint, 20m Sprint | Single-beam photocells (Globus Electronics, Codogne, Italy) |
| Lin et al. (2017) | Maximum Strength (Squat 1RM, Deadlift 1RM, Hip Thrust 1RM) | Olympic barbell, safety rack (Equipment not specified beyond type) |
|  | Vertical Jump (CMJ, SJ) | Yardstick |
|  | Speed (10m, 30m Sprint) | Electronic timing gates (Model not specified) |
|  | Agility T-Test | Electronic timing gates (Model not specified) |
| Zweifel et al. (2017) | Vertical Jump (CMJ) | Just Jump Mat (Probiotics) |
|  | Sprint Time (10y, 40y Dash) | Speed Trap I Timer (Power Systems, Inc.) |
|  | Broad Jump (BJ) | Standard measuring tape |
|  | Pro Agility Test (5-10-5) | Coach's Eye (TechSmith) |
|  | Estimated 1RM | Baechle and Earle tables |
