# Supplementary material for: Acute and long-term effects of hip thrust training on athletic performance: a systematic review and meta-analysis
Source: PeerJ. 2026 Feb 27;14:e20785. doi: 10.7717/peerj.20785 (PMC12951884; doi:10.7717/peerj.20785)
Supplement: Supplemental Information 6 [file peerj-14-20785-s006.docx]

**Title: Acute and Long-Term Effects of Hip Thrust Training on Athletic Performance: A Systematic Review and Meta-Analysis**

**Journal Name: *PeerJ***

**Authors:** Shengfa Lin^1^,Mengna Chen^1^,Xiaolan Yi^1^, Yuhao Li^1^, Ruidong Liu^1, 2,^*

**Affiliations:**

^1^ Sports Coaching College, Beijing Sport University, Haidian District, Beijing, China

^2^ Key Laboratory of Sport Training of General Administration of Sport of China, Beijing Sport University, Haidian District, Beijing, China

Corresponding Author:

Ruidong Liu

48 Xinxi Road, Haidian District, Beijing, 100084, China

Email address: lrd5156@bsu.edu.cn

**Supplementary file S1: Funnel plots.**


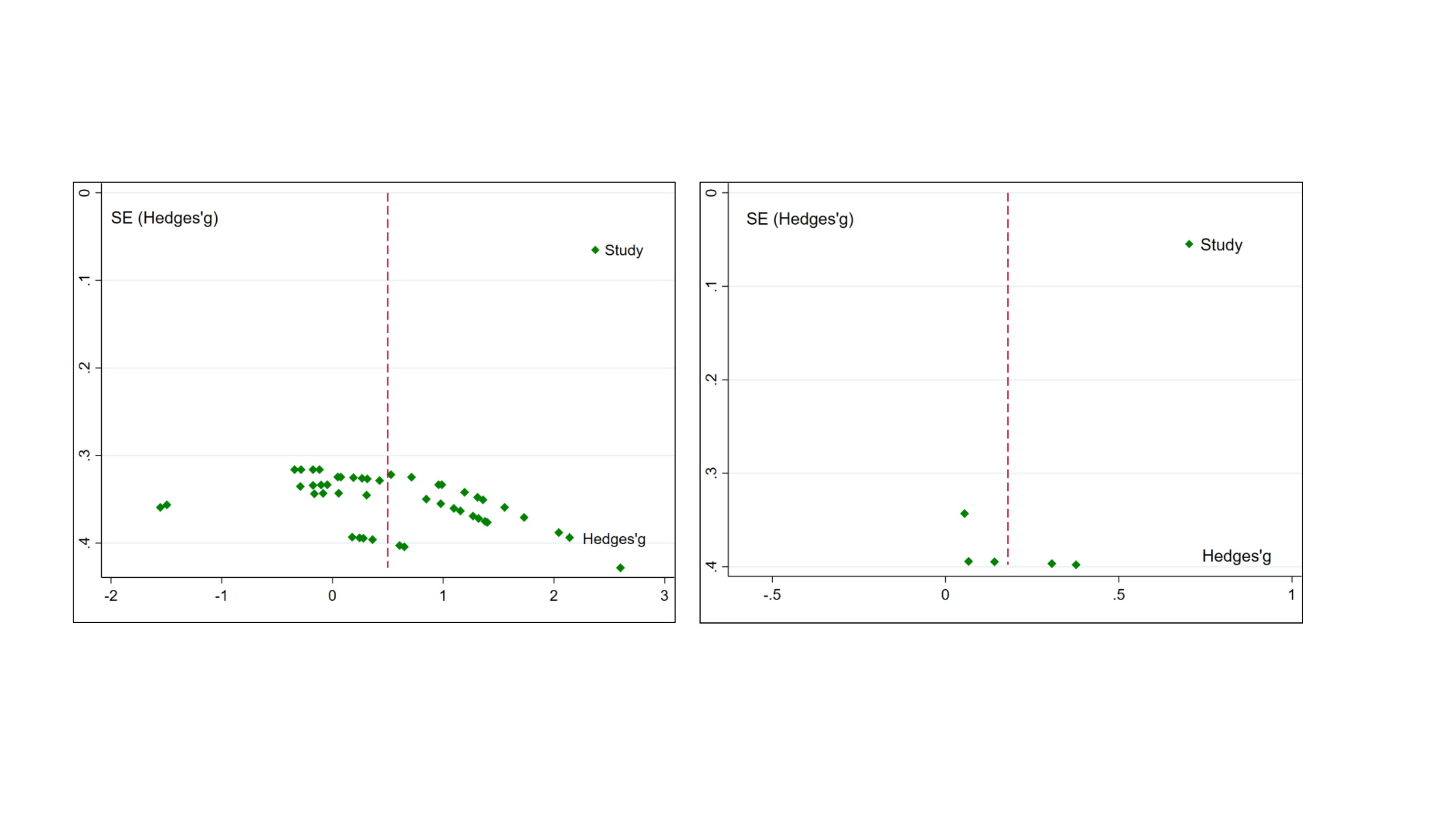


**Fig S1: Funnel plots for assessing publication bias for the following outcomes: left: acute linear acceleration sprint; right: acute jump performance.**


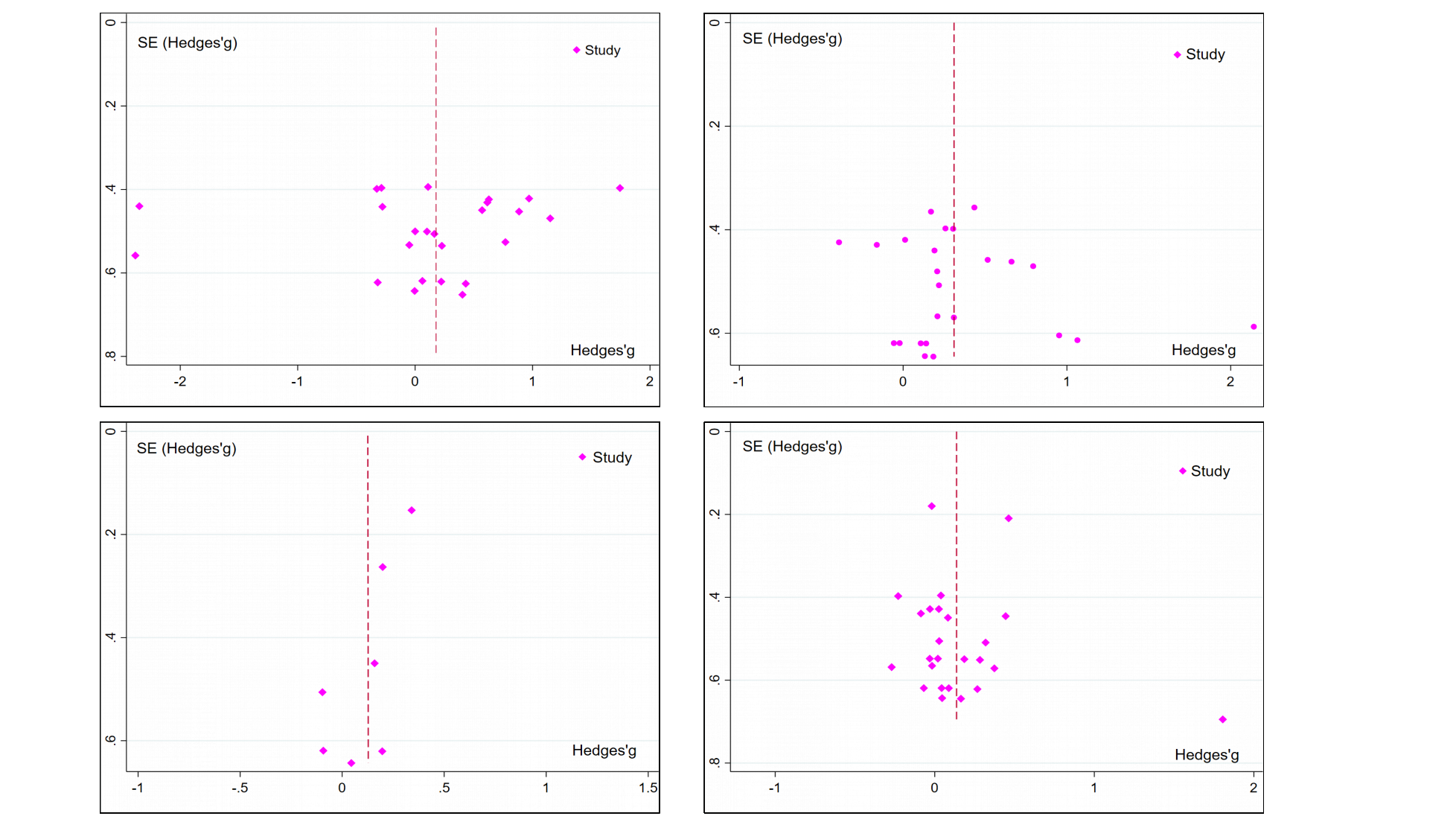


**Fig S2: Funnel plots for assessing publication bias for the following outcomes: top-left: long-term strength performance; top-right: linear acceleration sprint; bottom-left: change of direction performance; bottom-right: jump performance.**
