# Supplementary material for: Acute and long-term effects of hip thrust training on athletic performance: a systematic review and meta-analysis
Source: PeerJ. 2026 Feb 27;14:e20785. doi: 10.7717/peerj.20785 (PMC12951884; doi:10.7717/peerj.20785)
Supplement: Supplemental Information 7 [file peerj-14-20785-s007.docx]

| **Section and Topic** | **Item #** | **Checklist item** | **Location where item is reported** |
| --- | --- | --- | --- |
| **TITLE** | | |  |
| Title | 1 | Identify the report as a systematic review. | **Title,** "Acute and Long-Term Effects of Hip Thrust Training on Athletic Performance: A **Systematic Review and Meta-Analysis**". |
| **ABSTRACT** | | |  |
| Abstract | 2 | See the PRISMA 2020 for Abstracts checklist. | **Abstract Section,** A structured summary of the review is provided. |
| **INTRODUCTION** | | |  |
| Rationale | 3 | Describe the rationale for the review in the context of existing knowledge. | **Introduction Section,** "The transfer of gains from HT training to athletic performance remains the most debated and unresolved aspect of its efficacy"; "The existing literature presents a fragmented and often contradictory picture"; "...the field still lacks a definitive quantitative synthesis to resolve these controversies". |
| Objectives | 4 | Provide an explicit statement of the objective(s) or question(s) the review addresses. | **Introduction Section**, "...this study provides the first systematic review and meta-analysis to aggregate the current body of research, aiming to determine the acute and long-term effects of the HT exercise on measures of athletic performance, including strength, linear acceleration, change of direction (COD) speed, and jump performance" |
| **METHODS** | | |  |
| Eligibility criteria | 5 | Specify the inclusion and exclusion criteria for the review and how studies were grouped for the syntheses. | **Inclusion and Exclusion Criteria Section (PICOS framework)** |
| Information sources | 6 | Specify all databases, registers, websites, organisations, reference lists and other sources searched or consulted to identify studies. Specify the date when each source was last searched or consulted. | **Search Strategy Section**, "A comprehensive search was conducted across multiple electronic databases, including PubMed, Web of Science, Scopus, CINAHL, MEDLINE, and SPORTDiscus, from their inception up to August 10, 2025"; A manual search was also conducted, involving backward and forward citation searching of included articles and relevant reviews. |
| Search strategy | 7 | Present the full search strategies for all databases, registers and websites, including any filters and limits used. | **Search Strategy Section**, "The complete, database-specific search syntax is provided in the Supplemental Table S1" |
| Selection process | 8 | Specify the methods used to decide whether a study met the inclusion criteria of the review, including how many reviewers screened each record and each report retrieved, whether they worked independently, and if applicable, details of automation tools used in the process. | **Inclusion and Exclusion Criteria Section**, "Two of the authors (SL and MC) conducted the initial…disagreement, a third author (YL) was included in the review process to provide a consensus". |
| Data collection process | 9 | Specify the methods used to collect data from reports, including how many reviewers collected data from each report, whether they worked independently, any processes for obtaining or confirming data from study investigators, and if applicable, details of automation tools used in the process. | **Data Extraction Section,** "Two independent reviewers (SL and MC) systematically extracted…with a third reviewer (YL) to achieve consensus"; Software (WebPlotDigitizer) was used to extract data from graphs; Corresponding authors were contacted for missing or unclear data. |
| Data items | 10a | List and define all outcomes for which data were sought. Specify whether all results that were compatible with each outcome domain in each study were sought (e.g. for all measures, time points, analyses), and if not, the methods used to decide which results to collect. | **Outcomes & Data Extraction Sections**, Outcome data sought included the "mean and standard deviation (SD) of performance measures for all groups at pre- and post-intervention time points"; Defined outcomes were: "strength (e.g., SQ strength), speed (e.g., linear acceleration sprint time over a defined distance), COD speed... or jump performance". |
|  | 10b | List and define all other variables for which data were sought (e.g. participant and intervention characteristics, funding sources). Describe any assumptions made about any missing or unclear information. | **Data Extraction Section**, Other extracted variables included: "(1) study author and publication year; (2) participant characteristics (sample size, age, sex, sport background, and resistance training experience); (3) details of the acute intervention (volume, intensity, and rest interval duration); (4) details of the long-term intervention (training frequency, duration, volume, and intensity)" |
| Study risk of bias assessment | 11 | Specify the methods used to assess risk of bias in the included studies, including details of the tool(s) used, how many reviewers assessed each study and whether they worked independently, and if applicable, details of automation tools used in the process. | **Methodological Assessment Section**, "The methodological quality of each included study was independently assessed by two reviewers ([SL] and [MC]) using the Physiotherapy Evidence Database (PEDro) scale"; "Any discrepancies... were resolved through discussion and, if necessary, consultation with a third reviewer ([YL]) to reach a final consensus". |
| Effect measures | 12 | Specify for each outcome the effect measure(s) (e.g. risk ratio, mean difference) used in the synthesis or presentation of results. | **Meta-analysis and Risk of Bias Section**, "The effect size (ES) was calculated as Hedges' g, which provides a standardized mean difference (SMD) adjusted for small sample bias" |
| Synthesis methods | 13a | Describe the processes used to decide which studies were eligible for each synthesis (e.g. tabulating the study intervention characteristics and comparing against the planned groups for each synthesis (item #5)). | **Methods Section,** Eligibility for synthesis was determined by the PICOS criteria. Only studies providing sufficient data for effect size calculation were included in the quantitative synthesis (meta-analysis). |
|  | 13b | Describe any methods required to prepare the data for presentation or synthesis, such as handling of missing summary statistics, or data conversions. | **Data Extraction Section**, "If data were presented only in graphical format, specialized software (WebPlotDigitizer, version 5.2) was used to digitize and extract the numerical values"; "In cases of missing or incompletely reported data, the corresponding authors of the original studies were contacted for clarification". |
|  | 13c | Describe any methods used to tabulate or visually display results of individual studies and syntheses. | **Meta-analysis and Risk of Bias & Results Sections**, Funnel plots were used to assess publication bias ("All funnel plots are available in the Supplemental Articles S1"); Forest plots (Figures 2-7) were used to present the results of the meta-analyses. |
|  | 13d | Describe any methods used to synthesize results and provide a rationale for the choice(s). If meta-analysis was performed, describe the model(s), method(s) to identify the presence and extent of statistical heterogeneity, and software package(s) used. | **Meta-analysis and Risk of Bias Section**, "a random-effects model (DerSimonian and Laird) was employed for all meta-analyses to pool the ES estimates". The rationale was the "anticipated variability in study populations and intervention protocols"; The I² statistic was used to assess heterogeneity; Analyses were conducted using "Stata/MP (version 17.0...)" |
|  | 13e | Describe any methods used to explore possible causes of heterogeneity among study results (e.g. subgroup analysis, meta-regression). | **Moderator analysis Section,** "To investigate potential sources of heterogeneity, a series of pre-planned moderator analyses were conducted"; Moderators included recovery duration, training volume, age, sex, intervention duration, etc. |
|  | 13f | Describe any sensitivity analyses conducted to assess robustness of the synthesized results. | **Meta-analysis and Risk of Bias & Results Sections**, "In cases where significant bias was detected, a sensitivity analysis was conducted using the trim and fill method..."; A sensitivity analysis was conducted for SQ strength: "After a sensitivity analysis, excluding the study of (Barbalho et al. 2020), similar results were found...". |
| Reporting bias assessment | 14 | Describe any methods used to assess risk of bias due to missing results in a synthesis (arising from reporting biases). | **Meta-analysis and Risk of Bias Section**, "The risk of publication bias was evaluated for each outcome by visually inspecting the symmetry of its corresponding funnel plot... this visual assessment was supplemented with a formal statistical analysis using Egger's regression test". |
| Certainty assessment | 15 | Describe any methods used to assess certainty (or confidence) in the body of evidence for an outcome. | Certainty of Evidence |
| **RESULTS** | | |  |
| Study selection | 16a | Describe the results of the search and selection process, from the number of records identified in the search to the number of studies included in the review, ideally using a flow diagram. | **Study selection Section & Figure 1**, "The initial search yielded a total of 699 records... After the removal of 466 duplicate records, 233 unique articles proceeded to the title and abstract screening phase... 38 articles for full-text eligibility assessment... a total of 23 studies met the inclusion criteria"; "The detailed flowchart of the entire study selection process is presented in Figure 1". |
|  | 16b | Cite studies that might appear to meet the inclusion criteria, but which were excluded, and explain why they were excluded. | **Study selection Section,** 15 articles were excluded at the full-text review stage; "The primary reasons for exclusion at this stage were: reporting ineligible outcomes (n = 8), not utilizing a HT intervention (n = 2), absence of a control group (n = 3), ineligible publication type (n = 1), and publication not in English (n = 1)". |
| Study characteristics | 17 | Cite each included study and present its characteristics. | **Study characteristics Section & Tables 2 and 3,** "A total of 9 studies that investigated acute effects were identified (Table 2)"; "A total of 14 long-term studies were identified (Table 3)"; The section and tables detail the participant characteristics, intervention protocols, and outcomes for all included studies. |
| Risk of bias in studies | 18 | Present assessments of risk of bias for each included study. | **Methodological Assessment of included studies Section & Table 1,** "The overall quality of the included studies ranged from "fair" to "good", with total scores ranging from 4 to 7 out of a possible 10"; Strengths (e.g., random allocation) and weaknesses (e.g., lack of blinding) are discussed; "The complete PEDro score for each study is presented in Table 1" |
| Results of individual studies | 19 | For all outcomes, present, for each study: (a) summary statistics for each group (where appropriate) and (b) an effect estimate and its precision (e.g. confidence/credible interval), ideally using structured tables or plots. | **Results Section, Figures 2-7**, For each meta-analysis, the forest plots (Figures 2-7) present the effect estimate (Hedges' g) and 95% confidence interval for each individual study included in the synthesis. |
| Results of syntheses | 20a | For each synthesis, briefly summarise the characteristics and risk of bias among contributing studies. | **Study characteristics & Methodological Assessment Sections**, The characteristics and quality of the studies contributing to the syntheses are summarized in these sections and their corresponding tables (Tables 1, 2, and 3). |
|  | 20b | Present results of all statistical syntheses conducted. If meta-analysis was done, present for each the summary estimate and its precision (e.g. confidence/credible interval) and measures of statistical heterogeneity. If comparing groups, describe the direction of the effect. | **Meta-analysis of Acute... & Long-term... Sections** |
|  | 20c | Present results of all investigations of possible causes of heterogeneity among study results. | **Moderator analyses Section & Table 4,** "For the acute effects on linear acceleration sprint, significant differences were found between recovery durations (p < 0.001) and between training volumes (p = 0.03)."; "For long-term training adaptations, a significant difference was observed in linear acceleration sprint performance between age groups (p = 0.01), favoring the <18 years group". |
|  | 20d | Present results of all sensitivity analyses conducted to assess the robustness of the synthesized results. | **Results Section,** "a subsequent trim-and-fill analysis did not impute any ‘missing’ studies..."; "After a sensitivity analysis, excluding the study of (Barbalho et al. 2020), similar results were found (ES = -0.02; 95% CI = -0.32 to 0.28; p = 0.90; I2 = 0.0%)" |
| Reporting biases | 21 | Present assessments of risk of bias due to missing results (arising from reporting biases) for each synthesis assessed. | **Meta-analysis... Sections**, Assessments are reported for each outcome. For acute sprint: "Egger's test indicating significant publication bias (p = 0.001)". For long-term HT strength: "Egger's test indicating no significant publication bias (p = 0.54)". |
| Certainty of evidence | 22 | Present assessments of certainty (or confidence) in the body of evidence for each outcome assessed. | Certainty of Evidence |
| **DISCUSSION** | | |  |
| Discussion | 23a | Provide a general interpretation of the results in the context of other evidence. | **Discussion Section**, "The results of this meta-analysis serve to reconcile a foundational debate surrounding the HT... The main findings reveal a nuanced reality: the HT’s exceptional capacity for acute muscle activation... does not uniformly translate into superior performance..."; The results are interpreted in the context of the "force-vector theory," dynamic correspondence, and other relevant physiological principles |
|  | 23b | Discuss any limitations of the evidence included in the review. | **Limitations Section,** "...some included studies used the HT as the foundational strength exercise but also incorporated other auxiliary exercises, meaning the observed effects cannot be entirely attributed to the HT alone"; "...weaknesses remain, including small sample sizes and a near-universal lack of allocation concealment, increasing the risk of bias". |
|  | 23c | Discuss any limitations of the review processes used. | **Limitations Section,** "Third, significant heterogeneity was observed in the acute sprint analysis (I² = 81.50%)"; "Finally, the conclusions should be interpreted with caution due to the limited number of primary studies available for certain moderator analyses..." |
|  | 23d | Discuss implications of the results for practice, policy, and future research. | Practical Applications & Future Directions Sections. |
| **OTHER INFORMATION** | | |  |
| Registration and protocol | 24a | Provide registration information for the review, including register name and registration number, or state that the review was not registered. | **Materials & Methods Section**, "The study was registered in the Open Science Framework (OSF Registries) The registered document is accessible via the following link: https:// osf.io/t7dkj" |
|  | 24b | Indicate where the review protocol can be accessed, or state that a protocol was not prepared. | **Materials & Methods Section,** the protocol can be accessed via the OSF registration link: "https:// osf.io/t7dkj. |
|  | 24c | Describe and explain any amendments to information provided at registration or in the protocol. | Not Reported. |
| Support | 25 | Describe sources of financial or non-financial support for the review, and the role of the funders or sponsors in the review. | **Acknowledgements Section**, "No acknowledgments". This implies no sources of support to declare. |
| Competing interests | 26 | Declare any competing interests of review authors. | **Not Reported**. A competing interests section is not present in the manuscript. |
| Availability of data, code and other materials | 27 | Report which of the following are publicly available and where they can be found: template data collection forms; data extracted from included studies; data used for all analyses; analytic code; any other materials used in the review. | **Not Reported**. The manuscript does not include a statement on the availability of data, code, or other materials. |

*From:*  Page MJ, McKenzie JE, Bossuyt PM, Boutron I, Hoffmann TC, Mulrow CD, et al. The PRISMA 2020 statement: an updated guideline for reporting systematic reviews. BMJ 2021;372:n71. doi: 10.1136/bmj.n71
